# PaCRISPR: a server for predicting and visualizing anti-CRISPR proteins

**DOI:** 10.1093/nar/gkaa432

**Published:** 2020-05-27

**Authors:** Jiawei Wang, Wei Dai, Jiahui Li, Ruopeng Xie, Rhys A Dunstan, Christopher Stubenrauch, Yanju Zhang, Trevor Lithgow

**Affiliations:** Infection and Immunity Program, Biomedicine Discovery Institute and Department of Microbiology, Monash University, VIC 3800, Australia; Infection and Immunity Program, Biomedicine Discovery Institute and Department of Microbiology, Monash University, VIC 3800, Australia; School of Computer Science and Information Security, Guilin University of Electronic Technology, Guilin 541004, China; School of Computer Science and Information Security, Guilin University of Electronic Technology, Guilin 541004, China; School of Computer Science and Information Security, Guilin University of Electronic Technology, Guilin 541004, China; Infection and Immunity Program, Biomedicine Discovery Institute and Department of Microbiology, Monash University, VIC 3800, Australia; Infection and Immunity Program, Biomedicine Discovery Institute and Department of Microbiology, Monash University, VIC 3800, Australia; School of Computer Science and Information Security, Guilin University of Electronic Technology, Guilin 541004, China; Infection and Immunity Program, Biomedicine Discovery Institute and Department of Microbiology, Monash University, VIC 3800, Australia

## Abstract

Anti-CRISPRs are widespread amongst bacteriophage and promote bacteriophage infection by inactivating the bacterial host's CRISPR–Cas defence system. Identifying and characterizing anti-CRISPR proteins opens an avenue to explore and control CRISPR–Cas machineries for the development of new CRISPR–Cas based biotechnological and therapeutic tools. Past studies have identified anti-CRISPRs in several model phage genomes, but a challenge exists to comprehensively screen for anti-CRISPRs accurately and efficiently from genome and metagenome sequence data. Here, we have developed an ensemble learning based predictor, PaCRISPR, to accurately identify anti-CRISPRs from protein datasets derived from genome and metagenome sequencing projects. PaCRISPR employs different types of feature recognition united within an ensemble framework. Extensive cross-validation and independent tests show that PaCRISPR achieves a significantly more accurate performance compared with homology-based baseline predictors and an existing toolkit. The performance of PaCRISPR was further validated in discovering anti-CRISPRs that were not part of the training for PaCRISPR, but which were recently demonstrated to function as anti-CRISPRs for phage infections. Data visualization on anti-CRISPR relationships, highlighting sequence similarity and phylogenetic considerations, is part of the output from the PaCRISPR toolkit, which is freely available at http://pacrispr.erc.monash.edu/.

## INTRODUCTION

Bacteria protect themselves from bacteriophage (phage) infections through a variety of different mechanisms, including the CRISPR–Cas adaptive immune system and restriction modification systems. To counteract different CRISPR–Cas systems, phages have evolved protein inhibitors known as anti-CRISPRs (1–6). Identification of novel anti-CRISPR systems promises several downstream applications, such as gene editing technologies and phage therapy (5,7). There is a resurgence in interest in discovering and using phages on two fronts: for phage therapies to treat humans with drug-resistant bacterial infections (8), and for phage-based decontamination in the food-processing industry (9–11), but our capacity to use phage as products is hindered by gaps in our knowledge of the fundamental biology of how phages interact with their host bacteria (12). From within the growing number of anti-CRISPRs (13,14) are those demonstrated to inactivate different types of CRISPR–Cas systems in a diverse number of bacterial species (4,5,15–21). Given their widespread distribution (Supplementary Figures S1 and S2) and broad specificity (Supplementary Figures S3 and S4), it is speculated that for each CRISPR–Cas system there could be a dedicated anti-CRISPR available (5).

Several strategies have been used to identify anti-CRISPRs (3,5), including bioinformatic analyses such as the ‘Guilt by association’ (15) or self-targeting method (20), and functional assays or screens (1,16,18). While these approaches have successfully identified anti-CRISPRs, these studies identified only some subsets of anti-CRISPRs and were highly dependent on prior knowledge of the functional features of an individual phage-host relationship. Initially, BLAST-based searches to retrieve homologues of anti-CRISPRs from related phages helped to identify how widespread some anti-CRISPRs are (15,22). However, considering that some anti-CRISPRs recently discovered have no discernible sequence similarity to those currently known, homology-based methods alone cannot be relied upon to identify novel anti-CRISPRs types.

To address this issue, machine learning methods were introduced for more accurate anti-CRISPR predictions. Gussow *et al.* developed a random forest based model, which was fed with features, including protein length, whether it was annotated, and its mean hydrophobicity (doi: https://doi.org/10.1101/2020.01.23.916767). Using this model, a diverse array of anti-CRISPRs were predicted and made publicly accessible. While this warehouse stores many potential anti-CRISPRs for later experimental confirmation, it does not allow researchers to perform their own anti-CRISPR predictions. Eitzinger *et al.* developed an eXtreme Gradient Boosting based predictor AcRanker, and fed their model with features, including amino acid composition (AAC) and grouped dimer- and trimer-frequency counts based on the physicochemical properties of these amino acids (23). Ten candidates predicted by AcRanker led to the discovery of two previously unknown anti-CRISPRs, which were experimentally validated in the same work (23). The AcRanker toolkit enables scientists to directly rank potential anti-CRISPRs for a given phage proteome but doesn’t explicitly indicate their prediction score or likelihood of being an anti-CRISPR. We sought to develop a new, user-friendly web server with high prediction accuracy, detailed annotation information and graphic visualizations.

Here we present a machine learning based predictor, PaCRISPR, to efficiently and accurately identify anti-CRISPRs based on protein sequences. PaCRISPR extracts four types of evolutionary features to mine patterns and characteristics from an experimentally validated dataset and trains a set of baseline models with each of the features. PaCRISPR then integrates these baseline models to construct an ensemble model for final predictions. Extensive cross-validation, independent tests, and case studies demonstrated that PaCRISPR achieved a significantly higher performance in predicting anti-CRISPRs, when compared to the general homology-based baseline predictors and the existing predictor AcRanker. Implementing the ensemble model as a user-friendly web server, PaCRISPR not only provides easy-to-use anti-CRISPR prediction, but also provides interactive visualizations based on sequence similarity and phylogenetic analysis. The latter specifies the closest relationship with known anti-CRISPRs for each predicted anti-CRISPR, which could assist users in assigning annotations. In this way, PaCRISPR is expected to facilitate the discovery and characterisation of novel anti-CRISPRs from phage and bacterial proteomes, therefore promoting the discovery and understanding of principles behind phage-host interactions and the co-evolution of phages and bacteria.

## MATERIAL AND METHODS

**Figure 1.**
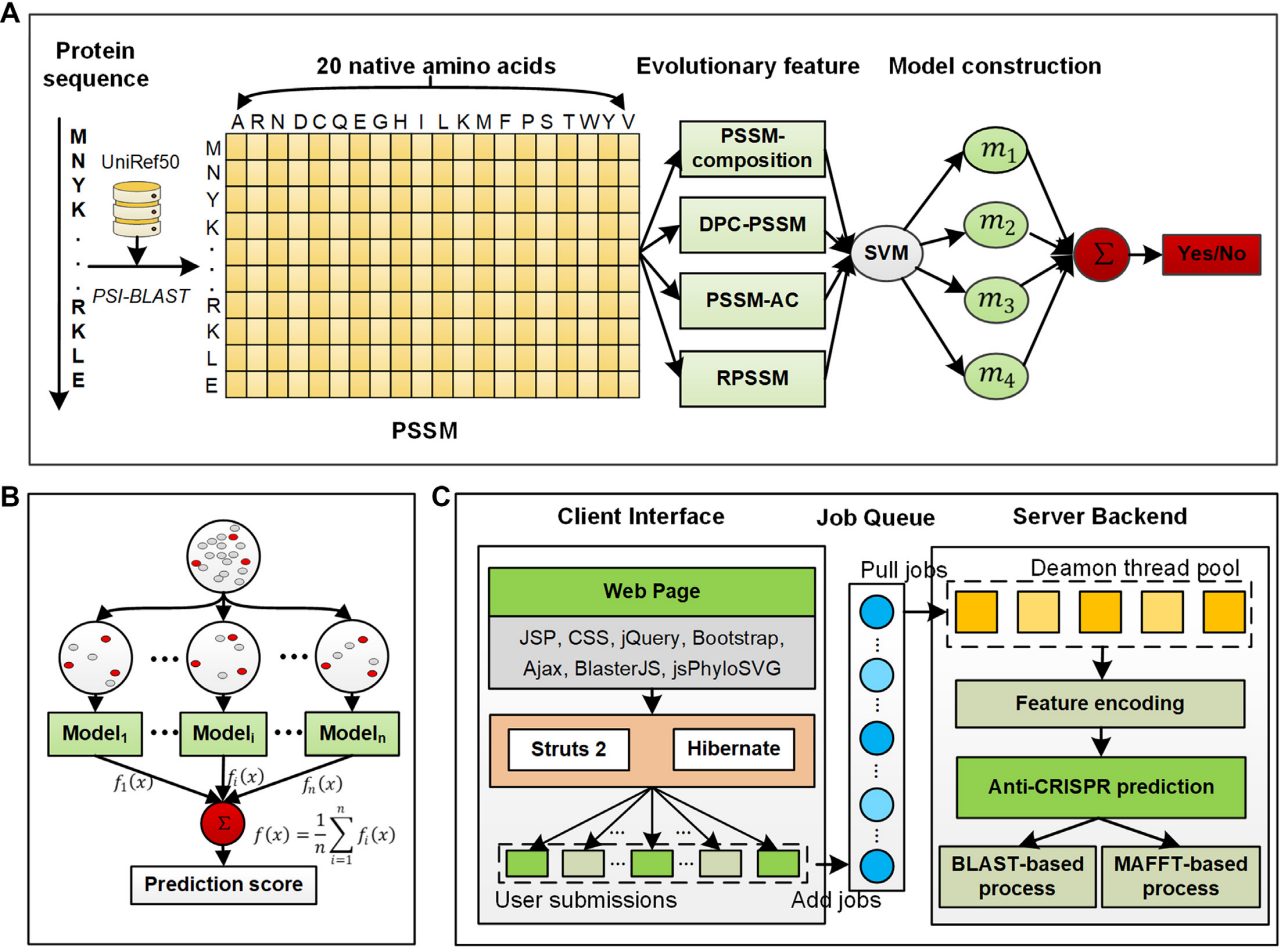
The methodology of the PaCRISPR server. (**A**) Ensemble model construction. (**B**) Multiple-time undersampling to solve the data imbalance problem. (**C**) The architecture of the PaCRISPR web server.

Here we describe the overall workflow of PaCRISPR in terms of data collection and curation, feature encoding, model training and integration, model performance evaluation, and toolkit development and usage (Figure 1).

### Data collection and curation

To train and test the proposed method, we extracted 488 experimentally validated anti-CRISPRs from the Anti-CRISPRdb (14) and from the literature (17,22). After removing redundant sequences with more than 70% sequence identity, we obtained 98 sequences as positive samples in the training dataset (Supplementary Table S1). Considering that anti-CRISPRs are small proteins and found from a limited set of phages, as well as from a limited set of mobile genetic elements (MGEs), we constructed negative samples in the training dataset with four strict criteria. Negative sample proteins: (i) must not be known or putative anti-CRISPRs themselves; (ii) must be isolated from phage or from bacterial MGEs (which may be known or putative MGEs), where the given bacterial genera are known to harbour anti-CRISPRs; (iii) must have <40% sequence similarity to each other and the 98 positive samples; (iv) must have lengths that fall in the range between 50 and 350 residues, which is similar in length to the 98 positive samples. In this way, we obtained a training dataset with 98 positive and 902 negative samples, and they have similar distributions (Supplementary Figure S5).

To further test the proposed method, 26 newly discovered, highly distinct anti-CRISPRs were subsequently collected from emerging papers that were recorded in the unified online anti-CRISPR resource (13). These 26 positive samples comprised the independent dataset, where they possess less than 10% similarity against the 98 anti-CRISPRs in the training dataset, except for two that have similarities of 21.38% and 56.12% (Supplementary Table S2). We then collected 260 non-anti-CRISPRs using similar criteria to the selection of negative samples in the training dataset, which have <40% sequence similarity against the training dataset and the positive samples in the independent dataset. In total, the independent dataset has 26 positive and 260 negative samples (Supplementary Figure S6).

As the predictor was trained with small proteins, it is necessary to test its predictive power when identifying long non-anti-CRISPRs. We constructed two pure negative datasets through retrieving 266 non-anti-CRISPRs from phages, and 597 non-anti-CRISPRs from bacterial known and putative MGEs. Both datasets have less than 40% sequence similarity against each other and the above datasets, and contain only sequences with length >=350 residues.

We additionally used 5 very recently discovered anti-CRISPRs and a bacterial contig as case studies to validate the prediction capability of the proposed method in a more practical scenario (Supplementary Table S3).

### Feature encoding

Novel anti-CRISPRs are especially difficult to identify given that they are significantly diverse, sharing no conserved sequence or structural motifs (3,4,24). The low sequence similarity therefore makes it particularly challenging to predict anti-CRISPRs from sequence-based features, which only mine characteristics from protein sequences. Instead, extracted from the Position-Specific Scoring Matrix (PSSM), evolutionary features to some extent track the evolutionary history of proteins and are proposed, therefore, to learn more informative patterns (25,26). Evolutionary features have been widely applied and demonstrated to have a significant contribution to protein attribute and function predictions, especially to identify those highly evolved proteins without observed signals (25–36).

To generate a PSSM, the PSI-BLAST program (37) (version blast-2.2.26 in this work) was used to iteratively (three iterations) search a given protein against a database (UniRef50) to detect its distantly related homologous proteins above a specified e-value score (0.001) (Figure 1A). Based on the multiple alignments of those homologous proteins, the generated PSSM combines their underlying conservation information and therefore could detect distant sequence similarities. For a protein with length of *L*, its PSSM is an *L* × 20 matrix (P = {${P_{i,j}}:i\ = \ 1 \ldots L;j\ = \ 1 \ldots 20$}), where 20 represents the number of native amino acid types (Figure 1A). The element ${P_{i,j}}$ is a score that indicates the conservation degree of the *j-*th amino acid type at the *i*th position of the protein sequence. A high score denotes a highly conserved position, while a low score denotes a weakly conserved position (25,38).

Here, using the POSSUM toolkit (39), we extracted four evolutionary features through mining information from PSSM in different aspects, including PSSM-composition (33), DPC-PSSM (35), PSSM-AC (36) and RPSSM (40) (explained below). We also implemented two commonly-used sequence-based features as baseline features, including the AAC and dipeptide composition (DPC). AAC counts the frequencies of residues, while DPC counts the frequencies of dipeptides in a protein sequence.

#### PSSM-composition

As the rows of a PSSM depend on the length of its protein sequence, PSSM-composition removes this variability by transforming the variably-sized PSSM into a fixed-size matrix. By summing up and averaging all rows for each native amino acid type, PSSM-composition transforms the original PSSM into a 20 × 20 matrix:

$$\begin{equation*}\ {R_i} = \frac{1}{L}\ \mathop \sum \limits_{k\ = \ 1}^L {r_k} \times \ {\delta _k}\end{equation*}$$

subject to

$$\begin{equation*}\left\{ {\begin{array}{@{}*{1}{c}@{}} {\ {\delta _k} = \ 1\ ,\ \ {p_k} = {a_i}\ }\\ {\ {\delta _k} = \ 0\ ,\ {p_k} \ne {a_i}} \end{array}} \right.\ \left( {i\ = \ 1, \ldots ,20;k\ = \ 1, \ldots ,L} \right)\end{equation*}$$

where ${R_i}$ represents the *i*th row of the resultant matrix, ${r_k}$ denotes the *k*th row of the PSSM, ${p_k}$ denotes the *k*th residue in the original protein sequence, and ${a_i}$ denotes the *i*th native type of amino acids. Finally, PSSM-composition converts the 20 × 20 matrix line-by-line into a single 400-dimensional vector.

#### DPC-PSSM

DPC-PSSM transforms the columns of the PSSM to mine its local sequence-order effect, and generates a 400-dimentional vector as follows:

$$\begin{equation*}{\rm{DPC}} - {\rm{PSSM\ }} = {\left( {{y_{1,1}}, \ldots ,{y_{1,20}},{y_{2,1}}, \ldots ,{y_{2,20}}, \ldots ,{y_{20,1}}, \ldots ,{\rm{\ }}{y_{20,20}}} \right)^T}\ \end{equation*}$$

subject to

$$\begin{equation*}\ {y_{i,j}} = \frac{1}{{L - 1}}\ \mathop \sum \limits_{k\ = \ 1}^{L - 1} {p_{k,i}} \times {p_{k + 1,j}}\ \left( {1 \le i,j \le 20} \right)\end{equation*}$$

where ${p_{k,i}}$ represents the element at *k*th row and *i*th column of the PSSM.

#### PSSM-AC

PSSM-AC calculates the correlation between two elements within the PSSM using the following formulas:

$$\begin{equation*}PSSM - AC \left( {j,lg} \right) = \mathop \sum \limits_{i = 1}^{L - lg} \frac{{\left( {{P_{i,j}} - \overline {{P_j}} } \right)\left( {{P_{i + lg,j}} - \overline {{P_j}} } \right)}}{{L - lg}}\ \end{equation*}$$

subject to

$$\begin{equation*}\ \overline {{P_j}} = \mathop \sum \limits_{i = 1}^L \frac{{{P_{i,j}}}}{L},\ j = 1, \ldots ,20\ \end{equation*}$$

where *lg* ranges from 1 to *LG*, and ${P_{i,j}}$ represents the element at *i*th row and *j*th column of the PSSM. As a result, the number of elements in the PSSM-AC vector amounts to 20 × *LG*, with *LG*<*L*. In this work, we used the default value 10 of the *LG*, and finally generated a 200-dimensional vector.

#### RPSSM

RPSSM explores the local sequence order effect but based on a reduced PSSM. It first generates an *L* × 10 reduced PSSM by merging some columns of the original PSSM, which could be represented as follows:

$$\begin{equation*}reduced - PSSM\ = \ \left( {{P_1}, \ldots ,{P_5}, \ldots ,{P_{10}}} \right)\end{equation*}$$

subject to

$$\begin{equation*}\begin{array}{@{}*{1}{l}@{}} {\ {P_1} = \frac{{{P_F} + {P_Y} + {P_W}}}{3},\quad {P_2} = \frac{{{P_M} + {P_L}}}{2},\quad {P_3} = \frac{{{P_I} + {P_V}}}{2},}\\ {{P_4} = \frac{{{P_A} + {P_T} + {P_S}}}{3},\quad {P_5} = \frac{{{P_N} + {P_H}}}{2},\quad {P_6} = \frac{{{P_Q} + {P_E} + {P_D}}}{3},}\\ {{P_7} = \frac{{{P_R} + {P_K}}}{2},\quad {P_8} = {P_C},\quad {P_9} = {P_G},\quad {P_{10}} = {P_P}} \end{array}\end{equation*}$$

where ${P_A}$, …, ${P_V}$ denote the 20 columns in the original PSSM corresponding to the 20 native types of amino acids. The reduced PSSM is further transformed into a 10-element vector:

$$\begin{equation*}\ {D_s} = \frac{{\mathop \sum \nolimits_{i = 1}^L {{\left( {{p_{i,s}} - \overline {{p_s}} } \right)}^2}}}{L}\ \end{equation*}$$

subject to

$$\begin{equation*}\ \overline {{p_s}} = \frac{1}{L}\ \mathop \sum \limits_{i\ = \ 1}^L {p_{i,s}},\ s\ = \ 1,2, \ldots ,10\end{equation*}$$

where ${p_{i,s}}$ represents the element at *i*th row and *s*th column of the reduced PSSM. Also, the reduced PSSM could be further transformed into a 10 × 10 matrix to explore its local sequence order effect:

$$\begin{equation*}\ {D_{s,t}} = \frac{1}{{L - 1}}\ \mathop \sum \limits_{i\ = \ 1}^{L - 1} {\chi _{i,i + 1}}\end{equation*}$$

subject to

$$\begin{equation*}\begin{array}{@{}*{3}{l}@{}} {\ {\chi _{i,i + 1}}}& = &{{{\left( {{p_{i,s}} - \frac{{{p_{i,s}} + {p_{i + 1,t}}}}{2}} \right)}^2}\ + \ {{\left( {{p_{i + 1,t}} - \frac{{{p_{i,s}} + {p_{i + 1,t}}}}{2}} \right)}^2}}\\ {}&{ = \ }&{\frac{{{{({p_{i,s}} - {p_{i + 1,t}})}^2}}}{2}\ ,\ s,t\ = \ 1,2, \ldots ,10.} \end{array}\end{equation*}$$

Finally, we obtained the RPSSM feature in 110 dimensions by combining ${D_{s,t}}$ and ${D_s}$.

$$\begin{equation*}RPSSM\ = \ \left[ {{D_{1,1}},{D_{1,2}}, \ldots ,{D_{10,10}},{D_1}, \ldots ,{D_{10}}} \right]\end{equation*}$$

### Model construction

To deal with the imbalanced classification problem, for each of the features, we constructed 10 subsets by combining the positive samples and the same numbers of randomly selected negative samples from the training datasets (Figure 1B). We accordingly trained 10 classifiers using the support vector machine (SVM) and integrated them by averaging their prediction outputs. SVM is widely used to solve binary classification problems in the field of computational biology (41). Particularly SVM with a radial basis function kernel (RBF) has been successfully used for nonlinear biological sequence classification (29,30). Two parameters affect the performance of the RBF kernel based SVM. Among them, Cost controls the cost of misclassification of data training, and Gamma is a specific parameter of the RBF kernel. In this study, for each SVM based classifier, the parameters Cost and Gamma were optimized using a grid search within the space {2^−10^,…,2^10^}. In this way, we obtained an ensemble model as the baseline model for each feature (termed single feature-based model) (30,32). To make full use of different types of evolutionary features, we averaged the prediction scores of their single feature-based models to form the final ensemble model (Figure 1A).

### Performance evaluation

The proposed method was rigorously and extensively validated based on the 5-fold cross-validation test, an additional independent test, and prediction capability was further investigated using case studies. Performance measurements include Sensitivity (SN), Specificity (SP), Accuracy (ACC), *F*-value and Matthews correlation coefficient (MCC) (42), which are defined as follows:

$$\begin{equation*}SN\ = \frac{{TP}}{{TP + FN}}\ \ \ \end{equation*}$$

$$\begin{equation*}SP\ = \frac{{TN}}{{TN + FP}}\ \ \end{equation*}$$

$$\begin{equation*}ACC\ = \frac{{TP + TN}}{{TP + FP + TN + FN}}{\rm{\ }}\end{equation*}$$

$$\begin{equation*}F - value\ = \ 2\ \times \frac{{TP}}{{2TP + FP + FN}}\end{equation*}$$

$$\begin{equation*}MCC\ = \frac{{\left( {TP \times TN} \right) - \left( {FN \times FP} \right)}}{{\sqrt {\left( {TP + FN} \right)\ \times \ \left( {TN + FP} \right)\ \times \ \left( {TP + FP} \right)\ \times \ \left( {TN + FN} \right)} }}\ \end{equation*}$$

where *TP*, *TN*, *FP* and *FN* denote the numbers of true positives, true negatives, false positives, and false negatives, respectively. For a predictor, SN and SP measure its power of identifying positive and negative samples, respectively. ACC, *F*-value, and MCC measure its comprehensive capability of identifying both positive and negative samples. Besides, the receiver operating characteristic (ROC) curve, with its AUC (area under the curve) value calculated, was used to visualize the prediction performance of a predictor.

### Server construction

The architecture of the PaCRISPR server consists of two components: a client web interface and a server backend (Figure 1C).

The client web interface is responsible for interacting with users through the input and output displays, and to process the service logic including the illegal character detection, sequence validation and format. The former was implemented by JSP, CSS, jQuery (https://jquery.com/), Bootstrap (https://bootstrapdocs.com/) and their extension packages. Specifically, the sequence similarity was visualized by BlasterJS (43), and the phylogenetic tree was presented using jsPhyloSVG (44).The latter was implemented by the JAVA (https://www.java.com/) server development suite, including Struts 2 (https://struts.apache.org/) and Hibernate (https://hibernate.org/).

The server backend is responsible for executing the whole prediction process, including encoding features, making predictions, and generating visualize-ready data. The prediction program was written in R language (https://www.r-project.org/) dependent on the e1071 package for SVM modelling (https://CRAN.R-project.org/package=e1071). The BLAST program (version 2.8.1+) (45) was used to search against the known anti-CRISPRs for each predicted anti-CRISPR, and to record regions of their similarities for sequence similarity visualization. The MAFFT toolkit (46) was used to generate multiple alignment results between each predicted anti-CRISPR and the known anti-CRISPRs for phylogenetic tree visualization. A Perl CGI (https://metacpan.org/pod/CGI) program was written to string together these steps within a single thread.

The client web interface interacts with the server backend through a fast and lightweight queueing system, implemented using the Gearman framework (http://gearman.org/). The client web interface simply puts the user's submissions (each of them as a job) into the queueing system, where the Perl idle threads, maintained in a daemon thread pool with customizable size, pull and execute the jobs. During the whole process, the MySQL database (https://www.mysql.com/) is used to store intermediate and final results, as well as synchronize messages between the client web interface and the server backend. In this way, the architecture brings better user experience by decoupling the client web interface that requires prompt response speed and the server backend that handles time-consuming jobs. This also makes the architecture amenable for expansions to add new computational facilities to meet the increasing demand in predicting ever accumulating genome-scale data.

## RESULTS

### Prediction performance evaluation and comparison

**Figure 2.**
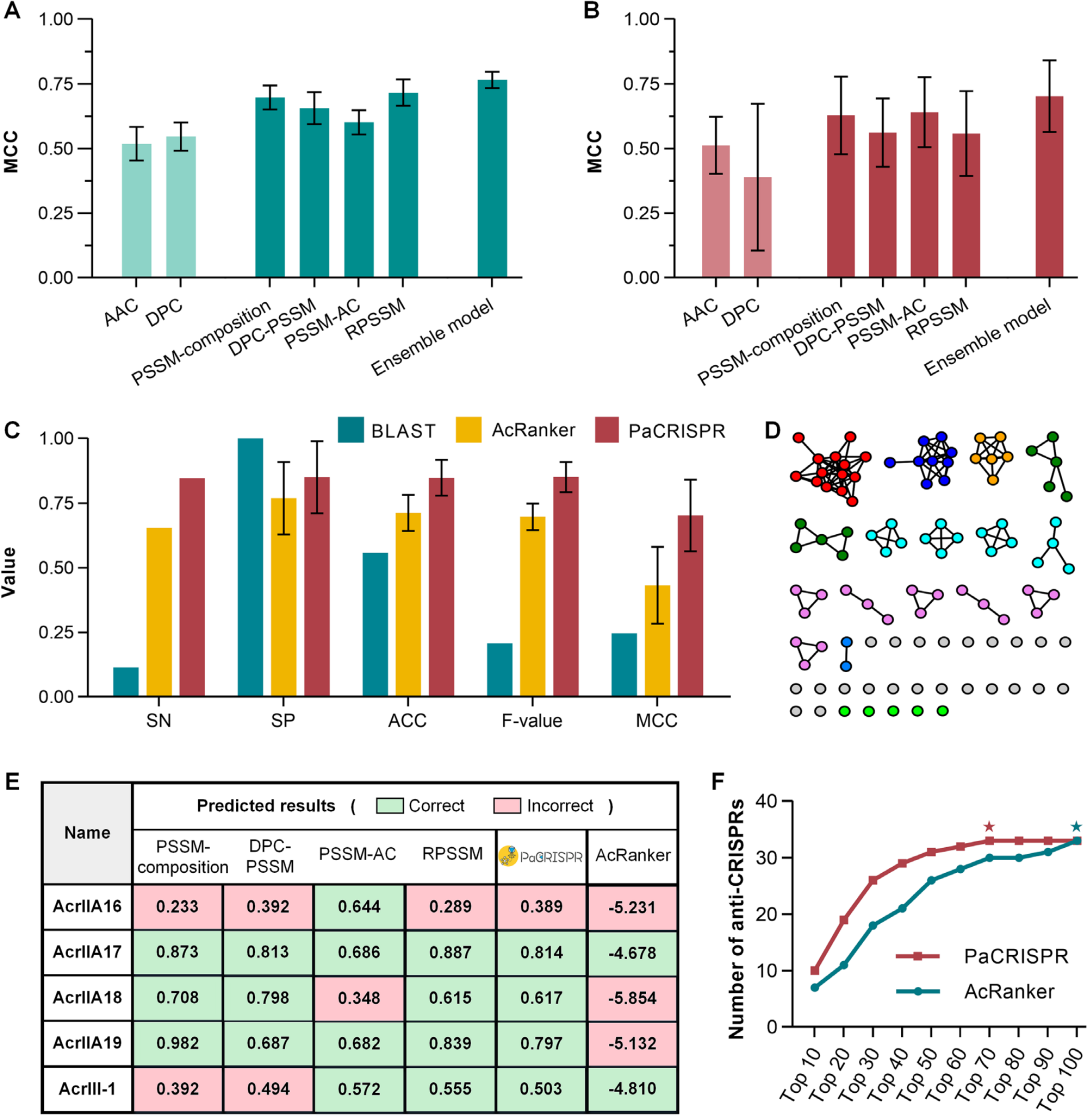
Performance evaluation and comparison. (**A**) Performance comparison between single feature-based models and the ensemble model based on the 5-fold cross-validation test. (**B**) Performance comparison between single feature-based models and the ensemble model based on the independent test. (**C**) Performance comparison between the PaCRISPR toolkit, homology-based baseline predictors and the AcRanker toolkit. (**D**) Sequence similarity network (SSN) based on the 98 anti-CRISPRs in the training dataset and 5 case study anti-CRISPRs. The SSN file was generated by conducting an all-against-all BLAST using the EFI-EST server (48), and visualized using the Cytoscape software (49). The five case-study anti-CRISPRs are represented by the final five green dots. (**E**) Prediction comparison of the case study anti-CRISPRs. (**F**) Prediction comparison of applications of predictors into a bacterial contig. Each point denotes the number of anti-CRISPRs retrieved within a specific ranking range. The red and green stars indicate the first ranking ranges of PaCRISPR and AcRanker where all 33 known anti-CRISPRs were retrieved, respectively.

We evaluated and compared the proposed method with its single evolutionary feature-based models, and two additional single sequence-based models using 5-fold cross-validation and independent tests. All 5-fold cross-validation tests were repeated based on N balanced training datasets (*N* = 10 in this work), and subsequently averaged as the final performance results. Similarly, all independent tests were conducted 10 times, each of which was executed based on a balanced independent dataset comprised of the 26 independent positive samples and 26 randomly chosen negative samples. All models adopted a default cut-off threshold of 0.5 to keep the balance of sensitivity and specificity. The final ensemble model of the PaCRISPR toolkit was obtained through integrating the four single evolutionary feature-based models. The PaCRISPR toolkit was additionally benchmarked with two homology-based baseline predictors and the AcRanker toolkit (http://acranker.pythonanywhere.com/) (23) using the independent dataset (Figure 2, Supplementary Figures S7–S9, and Tables S4–S8). The BLAST-based predictor was implemented based on the BLAST+ software (45). Each of query proteins was searched against positive samples in the training dataset with an *E*-value of 0.001 and predicted to be positive if there was a hit. The hidden Markov model (HMM)-based predictor was implemented based on the HMMER toolkit (47). The cut-off threshold of the AcRanker output was set to –5, optimized based on the independent dataset. A query protein with a prediction score of greater than –5 will be regarded as an anti-CRISPR.

In both test cases, similar observations were obtained (Figure 2A, B, Supplementary Tables S4–S6 and Figures S7–S8): (i) Models trained with evolutionary features achieved obviously superior performance in predicting anti-CRISPRs, as compared with another two sequence-based models. This situation commonly exists when predicting proteins that lack conserved domains or signals (28–30,32,33). (ii) Single feature-based models could not achieve stable prediction performance. The RPSSM-based model, with the best performance on the 5-fold cross-validation test (Figure 2A and Supplementary Table S4), reached the bottom of the PSSM-based models on the independent test (Figure 2B and Supplementary Table S5). In contrast, the worst model among PSSM-based models on the 5-fold cross-validation test (Figure 2A and Supplementary Table S4), i.e. PSSM-AC-based model, achieved the best prediction performance on the independent test (Figure 2B and Supplementary Table S5). (iii) The final ensemble model achieved a more stable and accurate prediction performance when compared to its single PSSM-based models on both 5-fold cross-validation and independent tests (Figure 2A, B, Supplementary Tables S4-S6 and Figures S7-S8). The highest ACC, *F*-value, MCC and AUC values reflect its comprehensive predictive power, while a balance of SN and SP (also the highest compared with others) demonstrated its capability in identifying both positive and negative samples.

All machine learning based models, including ours and the AcRanker, obviously outperformed the homology-based baseline predictors on the independent test, with a remarkably better recognition capability of anti-CRISPRs (Figure 2C, Supplementary Tables S6, S7 and Figure S9). The homology-based baseline predictors made a biased prediction, which tended to predict most of or all the independent samples to be negative. As the HMM-based predictor failed to recognize any anti-CRISPRs in the independent dataset, we excluded it when calculating those performance measurements. This demonstrated that simple sequence similarity-based methods were not sufficient to recognize anti-CRISPRs, as these proteins possess little sequence similarity (Figure 2D) due to rapid evolution. When compared with AcRanker, PaCRISPR is demonstrably more suitable for capturing the intrinsic patterns of non-homologous anti-CRISPRs because it extracts evolutionary features rather than relies primarily on sequence-based features that AcRanker uses.

When predicting long non-anti-CRISPR proteins from phages and bacterial MGEs, both PaCRISPR and AcRanker achieved high true negative prediction accuracy (Supplementary Table S8). Specifically, PaCRISPR and AcRanker correctly predict 258 and 265 non-anti-CRISPRs out of 266 phage proteins, and 596 and 595 non-anti-CRISPRs out of 597 bacterial proteins. This, together with the balance of high SN and SP values on both 5-fold cross-validation and independent tests, demonstrated the superior performance of PaCRISPR in identifying anti-CRISPRs from phage and bacterial proteomes with a low false positive rate.

### Case studies

Towards the end of this study, 5 new anti-CRISPR proteins were discovered, providing perfect case studies to compare the proposed method with its peers in terms of their generalized prediction capabilities. These proteins are significantly different from all anti-CRISPRs in the training dataset with less than 10% sequence similarity (Figure 2D and Supplementary Table S3).

Four anti-CRISPRs from AcrIIA16 to AcrIIA19 were found to inhibit the class II-A CRISPR–Cas systems across multiple host species (50). No single model except the PSSM-AC-based model could predict that AcrIIA16 is an anti-CRISPR, leading to the false prediction of PaCRISPR in this protein. PaCRISPR successfully identified the remaining 3 case study proteins to be anti-CRISPRs (Figure 2E). Among them, the successful hit for AcrIIA18 is direct evidence of the prediction stability of the proposed method, given the failure of one singe model to recognize this new anti-CRISPR.

AcrIII-1 (also known as the DUF1874 protein family) inhibits type III CRISPR systems (including both type III-A and III-B subtypes) and was identified in a range of viruses and plasmids from archaeal species (51). This makes AcrIII-1 taxonomically divergent from the anti-CRISPR proteins relevant in bacterial species and, unlike other anti-CRISPRs that function on specific CRISPR effector proteins, AcrIII-1 targets cyclic tetra-adenylate (cA4). Despite these unique differences, our proposed method successfully predicted it with a score of 0.503 (Figure 2E). Among the single models, the two trained with PSSM-AC and RPSSM predicted AcrIII-1 as an anti-CRISPR, while the others failed. This further highlights the robustness of the final ensemble model, and illustrates that discoveries are possible using PaCRISPR.

In summary, PaCRISPR (and its single models trained with PSSM-AC and RPSSM) successfully identified 4 out of 5 case study anti-CRISPRs. In contrast, both BLAST-based and HMM-based predictors failed to predict any of the newly identified proteins as anti-CRISPRs (data not shown). Both models rely on sequence similarity-based comparisons between the query proteins and previously known anti-CRISPRs, and in the case of each of the newly identified anti-CRISPR proteins above, the candidates show very low similarities with previously known anti-CRISPRs. Using the same threshold of –5, as used for the independent test, AcRanker picked out only AcrIIA17 and AcrIII-1 as anti-CRISPRs. However, the low prediction scores it gives to these new proteins would lead to a low rank in the context of genome-scale prediction.

We also sought to provide a sense of the false discovery rate, and indicate how obvious true discoveries would be in a ranked list from a model dataset. To this end, we established a model system consisting of a contig (NCBI-RefSeq: NZ_ALTM01000002.1) from the genome sequence of *Streptococcus agalactiae* strain GB00548. This contig encodes 93 proteins, only one of which is an anti-CRISPR, the novel AcrIIA21 discovered by AcRanker (23). We then added 32 known anti-CRISPRs to the contig: the sequence for AcrIIA20 (another AcRanker-discovered anti-CRISPR) and all the 26 independent and five case study anti-CRISPRs to construct a test set totalling 125 protein sequences. The 33 known anti-CRISPRs act as markers to measure the performance of the predictors in a practical scenario of screening genomic sequence data. The prediction results generated by PaCRISPR and AcRanker were ranked and listed in Figure 2F and Supplementary Tables S9-S10.

Compared to AcRanker, PaCRISPR generally ranked known anti-CRISPRs in higher ranking positions (Supplementary Table S9). For each ranking range (such as top 10, top 20 and likewise), PaCRISPR retrieved more anti-CRISPRs (Figure 2F and Supplementary Table S10). Specially, the lowest-ranking anti-CRISPR (AcrIIA21) predicted by PaCRISPR stayed in 64th place, while that (AcrVA4) predicted by AcRanker ranked 94th (Supplementary Table S9). These observations suggest a lower false positive rate of PaCRISPR, which would retrieve fewer false anti-CRISPRs to obtain the same number of genuine anti-CRISPRs in practical use.

### The PaCRISPR server

**Figure 3.**
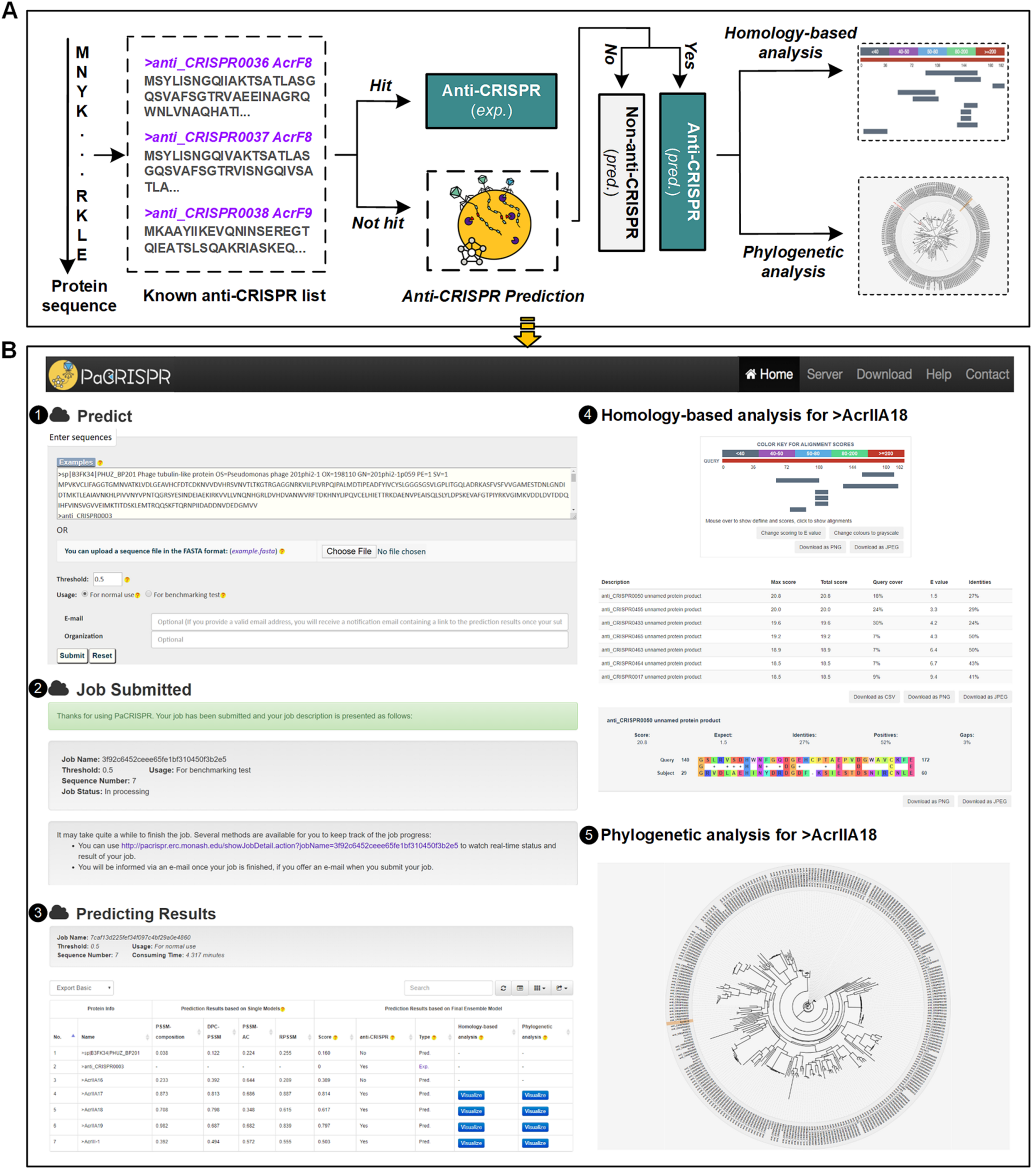
Demonstration of the PaCRISPR server. (**A**) The intuitive graphical workflow. (**B**) An example to showcase the usage of the PaCRISPR server from (1) the input page, (2) to the job summary page, (3) to the prediction results and (4–5) interactive visualizations.

PaCRISPR is a publicly available and user-friendly web server for predicting anti-CRISPRs and analysing their relationships with known anti-CRISPRs (Figure 3A). PaCRISPR allows users to copy-and-paste or upload their interested sequences in FASTA format in the input page (Figure 3B-1). When predicting query proteins, the PaCRISPR server provides two options in the Usage bar for different purposes. For normal use, the PaCRISPR server applies a built-in list of experimentally validated anti-CRISPRs to filter out the query proteins prior to using its models to execute the computational prediction. But for users who want to benchmark and test the prediction performance of the PaCRISPR server, they can disable the built-in list by selecting the ‘For benchmarking test’ option to retrieve the prediction scores for all query protein sequences. Once submitted, a unique link will be generated to refer to the job summary page (Figure 3B-2) during the job execution process. Users could use this link to track their job execution progress, and access or download their prediction results once completed (Figure 3B-3).

To distinguish between predicted and known anti-CRISPRs, each of the proteins will be marked with either an ‘Exp.’ if it has been experimentally validated or ‘Pred.’ with a prediction score. For validated anti-CRISPRs, users will be provided with a link to the relevant page in either the Anti-CRISPRdb (14) or the unified online anti-CRISPR resource (13) (Figure 3B-3). To facilitate downstream analysis, for each predicted anti-CRISPR, PaCRISPR lists the most homologous known anti-CRISPRs, charts their alignments, and highlights the pair-wise alignment (Figure 3B-4). The interactive phylogenetic tree can instead illustrate its relationships to known anti-CRISPRs with the predicted anti-CRISPR highlighted. Each of known anti-CRISPRs among the tree is marked with an external link to the Anti-CRISPRdb or the unified online anti-CRISPR resource, through which more detailed information could be obtained (Figure 3B-5).

## DISCUSSION

Discovery of new anti-CRISPRs opens an avenue for manipulating CRISPR–Cas machineries as a tool in gene editing or gene therapy, and provides new insights into phage interactions with their bacterial hosts. In this work, we have presented PaCRISPR, an ensemble and universal predictor to accurately and efficiently identify anti-CRISPRs from genome- and metagenome-derived sequence data. PaCRISPR aims at mining and extracting distinctive patterns and characteristics from known anti-CRISPRs by incorporating multiple evolutionary features within an ensemble framework. Compared with sequence-based features, the evolutionary features were demonstrated more suitable to predict this type of highly evolutionary proteins. Having been extensively and rigorously benchmarked in terms of prediction accuracy and robustness, PaCRISPR can identify novel anti-CRISPRs and outperforms other toolkits with a significant performance improvement. PaCRISPR differentiates itself with an extended interactive environment where users can tentatively annotate the putative anti-CRISPR based on the sequence similarity and phylogenetic analysis. It is anticipated that PaCRISPR could serve as a useful preliminary screening toolkit to identify potential anti-CRISPRs, and therefore expedite the discovery of novel anti-CRISPRs for their subsequent experimental validation. To ensure the PaCRISPR toolkit remains competitive and up-to-date, it will be periodically upgraded as new anti-CRISPRs are identified and experimentally validated.

## DATA AVAILABILITY

The PaCRISPR toolkit is freely available at http://pacrispr.erc.monash.edu/. All datasets used in this study have been uploaded to the Supplementary Data at NAR online and can also be downloaded via http://pacrispr.erc.monash.edu/download.jsp.

## Supplementary Material

gkaa432_Supplemental_Files
