# Supplementary material for: PaCRISPR: a server for predicting and visualizing anti-CRISPR proteins
Source: Nucleic Acids Res. 2020 May 27;48(W1):W348–57. doi: 10.1093/nar/gkaa432 (PMC7319593; doi:10.1093/nar/gkaa432)
Supplement: gkaa432_Supplemental_Files [file gkaa432_supplemental_files.zip › PaCRISPR_Supplementary_material.pdf]

PaCRISPR: a server for predicting and visualizing anti-CRISPR proteins

Supplementary Material

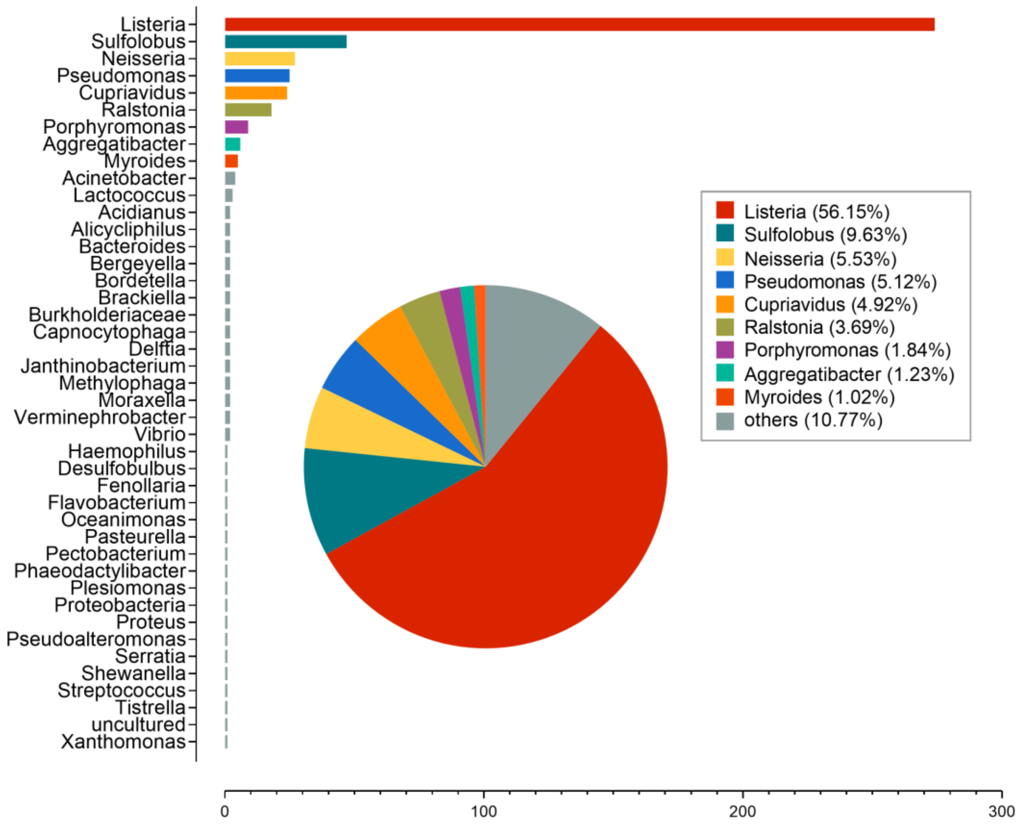

Fig. S1. Distribution of 488 anti-CRISPRs in terms of their bacterial origins based on the training dataset before redundant proteins were removed. The histogram chart counts the exact numbers of anti-CRISPRs among 43 genera. The pie chart shows the percentages of major anti-CRISPR associated genera.

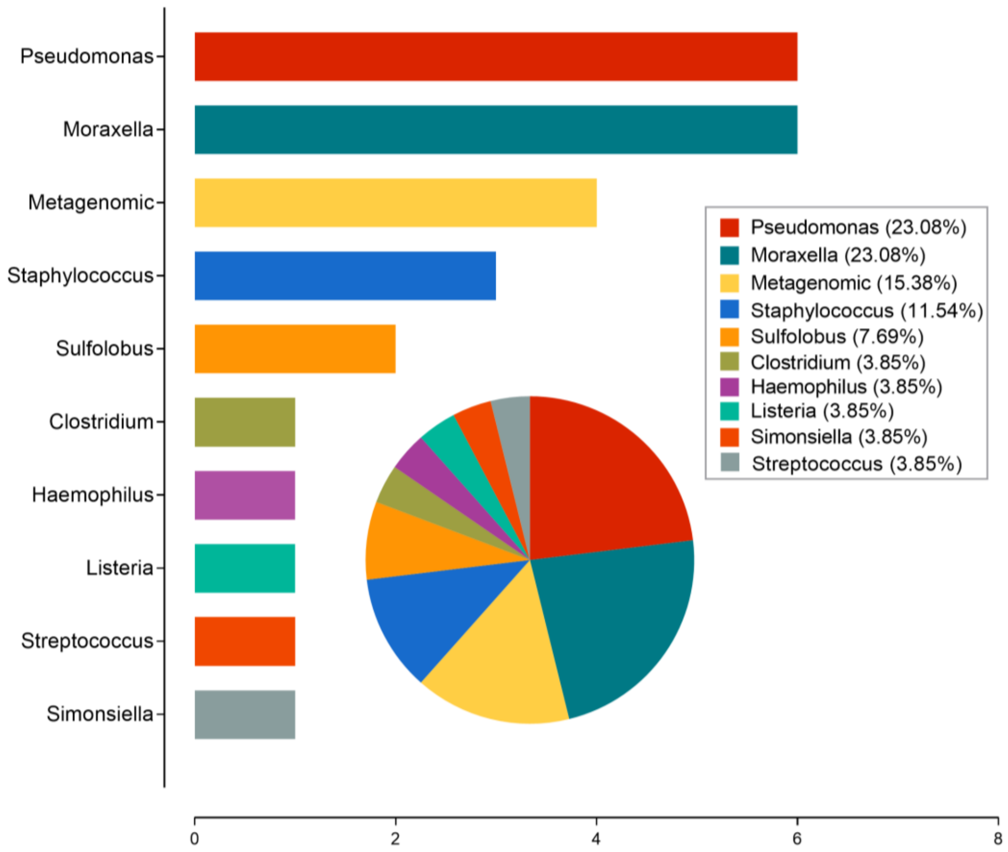

Fig. S2. Distribution of the 26 anti-CRISPRs comprising the independent dataset in terms of their bacterial origins. The histogram chart counts the exact numbers of anti-CRISPRs among 10 genera. The pie chart shows the percentages of whole anti-CRISPR associated genera.

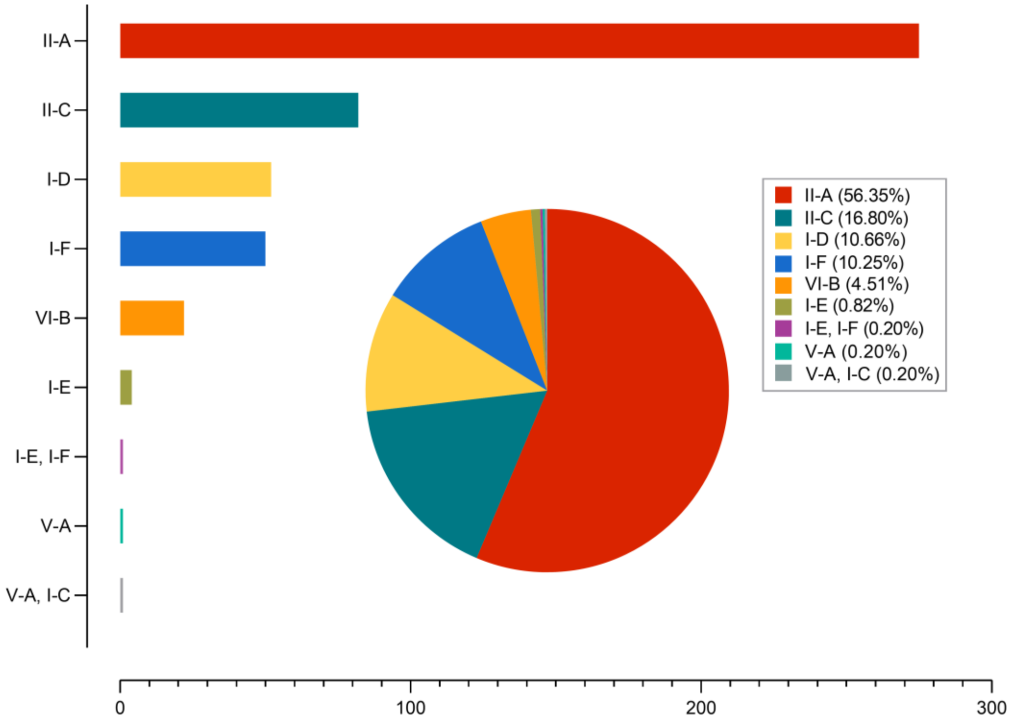

Fig. S3. Distribution of 488 anti-CRISPRs in terms of their inhibited types based on the training dataset before redundant proteins were removed. The histogram chart counts the exact numbers of 9 anti-CRISPR inhibiting types. The pie chart shows the percentage of each anti-CRISPR inhibiting type.

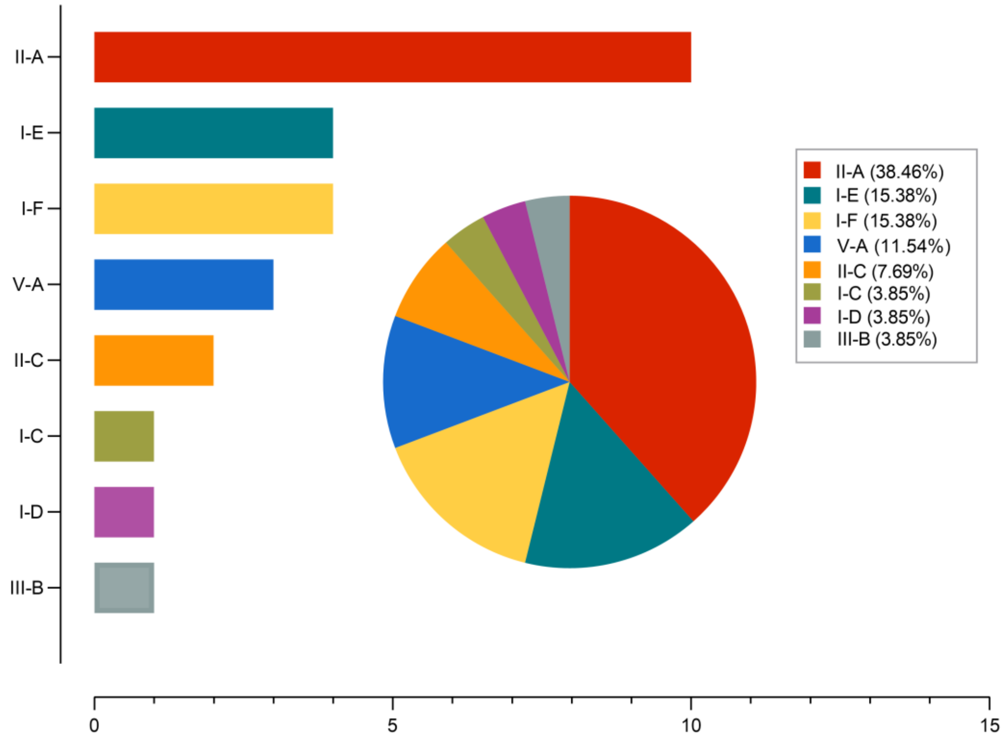

Fig. S4. Distribution of the 26 anti-CRISPRs comprising the independent dataset in terms of their inhibited types. The histogram chart counts the exact numbers of 8 anti-CRISPR inhibiting types. The pie chart shows the percentage of each anti-CRISPR inhibiting type.

Table S1. Details of all 98 anti-CRISPRs used in the training dataset.

ID	Name	Associated anti-CRISPR type	Bacterial origin	Reference
1	anti_CRISPR0001	I-F	*Pseudomonas*	(Bondy-Denomy, et al., 2013)
2	anti_CRISPR0002	I-F	*Pseudomonas*	(Bondy-Denomy, et al., 2013)
3	anti_CRISPR0003	I-F	*Pseudomonas*	(Bondy-Denomy, et al., 2013)
4	anti_CRISPR0005	I-F	*Pseudomonas*	(Bondy-Denomy, et al., 2013)
5	anti_CRISPR0007	I-F	*Pseudomonas*	(Bondy-Denomy, et al., 2013)
6	anti_CRISPR0008	I-E I-F	*Pseudomonas*	(Pawluk, et al., 2016)
7	anti_CRISPR0011	I-F	*Oceanimonas*	(Pawluk, et al., 2016)
8	anti_CRISPR0012	I-F	*Methylophaga*	(Pawluk, et al., 2016)
9	anti_CRISPR0013	I-F	*Methylophaga*	(Pawluk, et al., 2016)
10	anti_CRISPR0014	I-F	*Plesiomonas*	(Pawluk, et al., 2016)
11	anti_CRISPR0015	I-F	*Acinetobacter*	(Pawluk, et al., 2016)
12	anti_CRISPR0016	I-F	*Pasteurella*	(Pawluk, et al., 2016)
13	anti_CRISPR0017	I-F	*Acinetobacter*	(Pawluk, et al., 2016)
14	anti_CRISPR0019	I-F	*Proteobacteria*	(Pawluk, et al., 2016)
15	anti_CRISPR0021	I-F	*Pseudomonas*	(Pawluk, et al., 2016)
16	anti_CRISPR0030	I-F	*Pseudomonas*	(Pawluk, et al., 2016)
17	anti_CRISPR0032	I-F	*Janthinobacterium*	(Pawluk, et al., 2016)
18	anti_CRISPR0034	I-F	*Pectobacterium*	(Pawluk, et al., 2016)
19	anti_CRISPR0035	I-F	*Serratia*	(Pawluk, et al., 2016)
20	anti_CRISPR0036	I-F	*Delftia*	(Pawluk, et al., 2016)
21	anti_CRISPR0038	I-F	*Vibrio*	(Pawluk, et al., 2016)
22	anti_CRISPR0040	I-F	*Aggregatibacter*	(Pawluk, et al., 2016)
23	anti_CRISPR0042	I-F	*uncultured*	(Pawluk, et al., 2016)
24	anti_CRISPR0047	I-F	*Haemophilus*	(Pawluk, et al., 2016)
25	anti_CRISPR0048	I-F	*Desulfobulbus*	(Pawluk, et al., 2016)
26	anti_CRISPR0049	I-F	*Xanthomonas*	(Pawluk, et al., 2016)
27	anti_CRISPR0050	I-F	*Vibrio*	(Pawluk, et al., 2016)

28	anti_CRISPR0052	II-C	*Brackiella*	(Pawluk, et al., 2016)
29	anti_CRISPR0053	II-C	*Alicycliphilus*	(Pawluk, et al., 2016)
30	anti_CRISPR0055	II-C	*Bordetella*	(Pawluk, et al., 2016)
31	anti_CRISPR0057	II-C	*Alicycliphilus*	(Pawluk, et al., 2016)
32	anti_CRISPR0058	II-C	*Verminephrobacter*	(Pawluk, et al., 2016)
33	anti_CRISPR0059	II-C	*Pseudoalteromonas*	(Pawluk, et al., 2016)
34	anti_CRISPR0060	II-C	*Tistrella*	(Pawluk, et al., 2016)
35	anti_CRISPR0061	II-C	*Fenollaria*	(Pawluk, et al., 2016)
36	anti_CRISPR0062	II-C	*Bordetella*	(Pawluk, et al., 2016)
37	anti_CRISPR0064	II-C	*Neisseria*	(Pawluk, et al., 2016)
38	anti_CRISPR0086	II-C	*Ralstonia*	(Pawluk, et al., 2016)
39	anti_CRISPR0116	II-C	*Cupriavidus*	(Pawluk, et al., 2016)
40	anti_CRISPR0118	II-C	*Ralstonia*	(Pawluk, et al., 2016)
41	anti_CRISPR0127	II-C	*Neisseria*	(Pawluk, et al., 2016)
42	anti_CRISPR0132	II-C	*Neisseria*	(Pawluk, et al., 2016)
43	anti_CRISPR0133	II-A	*Listeria*	(Rauch, et al., 2017)
44	anti_CRISPR0245	II-A	*Listeria*	(Rauch, et al., 2017)
45	anti_CRISPR0253	II-A	*Listeria*	(Rauch, et al., 2017)
46	anti_CRISPR0275	II-A	*Listeria*	(Rauch, et al., 2017)
47	anti_CRISPR0331	II-A	*Listeria*	(Rauch, et al., 2017)
48	anti_CRISPR0384	II-A	*Listeria*	(Rauch, et al., 2017)
49	anti_CRISPR0407	I-E	*Pseudomonas*	(Pawluk, et al., 2014)
50	anti_CRISPR0408	I-E	*Pseudomonas*	(Pawluk, et al., 2014)
51	anti_CRISPR0409	I-E	*Pseudomonas*	(Pawluk, et al., 2014)
52	anti_CRISPR0410	I-E	*Pseudomonas*	(Pawluk, et al., 2014)
53	anti_CRISPR0411	VI-B	*Capnocytophaga*	(Smargon, et al., 2017)
54	anti_CRISPR0412	VI-B	*Phaeodactylibacter*	(Smargon, et al., 2017)
55	anti_CRISPR0413	VI-B	*Porphyromonas*	(Smargon, et al., 2017)
56	anti_CRISPR0422	VI-B	*Bacteroides*	(Smargon, et al., 2017)

57	anti_CRISPR0424	VI-B	*Flavobacterium*	(Smargon, et al., 2017)
58	anti_CRISPR0426	VI-B	*Myroides*	(Smargon, et al., 2017)
59	anti_CRISPR0430	VI-B	*Bergeyella*	(Smargon, et al., 2017)
60	anti_CRISPR0433	II-A	*Streptococcus*	(Hynes, et al., 2017)
61	anti_CRISPR0435	I-D	*Sulfolobus*	(He, et al., 2018)
62	anti_CRISPR0436	I-D	*Lactococcus*	(He, et al., 2018)
63	anti_CRISPR0439	I-D	*Sulfolobus*	(He, et al., 2018)
64	anti_CRISPR0440	I-D	*Sulfolobus*	(He, et al., 2018)
65	anti_CRISPR0441	I-D	*Sulfolobus*	(He, et al., 2018)
66	anti_CRISPR0442	I-D	*Sulfolobus*	(He, et al., 2018)
67	anti_CRISPR0443	I-D	*Sulfolobus*	(He, et al., 2018)
68	anti_CRISPR0445	I-D	*Sulfolobus*	(He, et al., 2018)
69	anti_CRISPR0446	I-D	*Sulfolobus*	(He, et al., 2018)
70	anti_CRISPR0447	I-D	*Sulfolobus*	(He, et al., 2018)
71	anti_CRISPR0448	I-D	*Sulfolobus*	(He, et al., 2018)
72	anti_CRISPR0449	I-D	*Sulfolobus*	(He, et al., 2018)
73	anti_CRISPR0450	I-D	*Sulfolobus*	(He, et al., 2018)
74	anti_CRISPR0451	I-D	*Sulfolobus*	(He, et al., 2018)
75	anti_CRISPR0452	I-D	*Sulfolobus*	(He, et al., 2018)
76	anti_CRISPR0453	I-D	*Sulfolobus*	(He, et al., 2018)
77	anti_CRISPR0454	I-D	*Acidianus*	(He, et al., 2018)
78	anti_CRISPR0455	I-D	*Sulfolobus*	(He, et al., 2018)
79	anti_CRISPR0456	I-D	*Acidianus*	(He, et al., 2018)
80	anti_CRISPR0457	I-D	*Sulfolobus*	(He, et al., 2018)
81	anti_CRISPR0458	I-D	*Sulfolobus*	(He, et al., 2018)
82	anti_CRISPR0460	I-D	*Sulfolobus*	(He, et al., 2018)
83	anti_CRISPR0461	I-D	*Sulfolobus*	(He, et al., 2018)
84	anti_CRISPR0462	I-D	*Sulfolobus*	(He, et al., 2018)
85	anti_CRISPR0463	I-D	*Sulfolobus*	(He, et al., 2018)

86	anti_CRISPR0467	I-D	*Sulfolobus*	(He, et al., 2018)
87	anti_CRISPR0468	I-D	*Sulfolobus*	(He, et al., 2018)
88	anti_CRISPR0471	I-D	*Sulfolobus*	(He, et al., 2018)
89	anti_CRISPR0472	I-D	*Sulfolobus*	(He, et al., 2018)
90	anti_CRISPR0475	I-D	*Sulfolobus*	(He, et al., 2018)
91	anti_CRISPR0476	I-D	*Sulfolobus*	(He, et al., 2018)
92	anti_CRISPR0477	I-D	*Sulfolobus*	(He, et al., 2018)
93	anti_CRISPR0478	I-D	*Sulfolobus*	(He, et al., 2018)
94	anti_CRISPR0482	I-D	*Sulfolobus*	(He, et al., 2018)
95	anti_CRISPR0484	I-D	*Sulfolobus*	(He, et al., 2018)
96	AcrIIC1	I-C	*Brackiella*	(Pawluk, et al., 2016)
97	AcrVA2	V-A	*Moraxella*	(Marino, et al., 2018)
98	AcrVA3	V-A I-C	*Moraxella*	(Marino, et al., 2018)

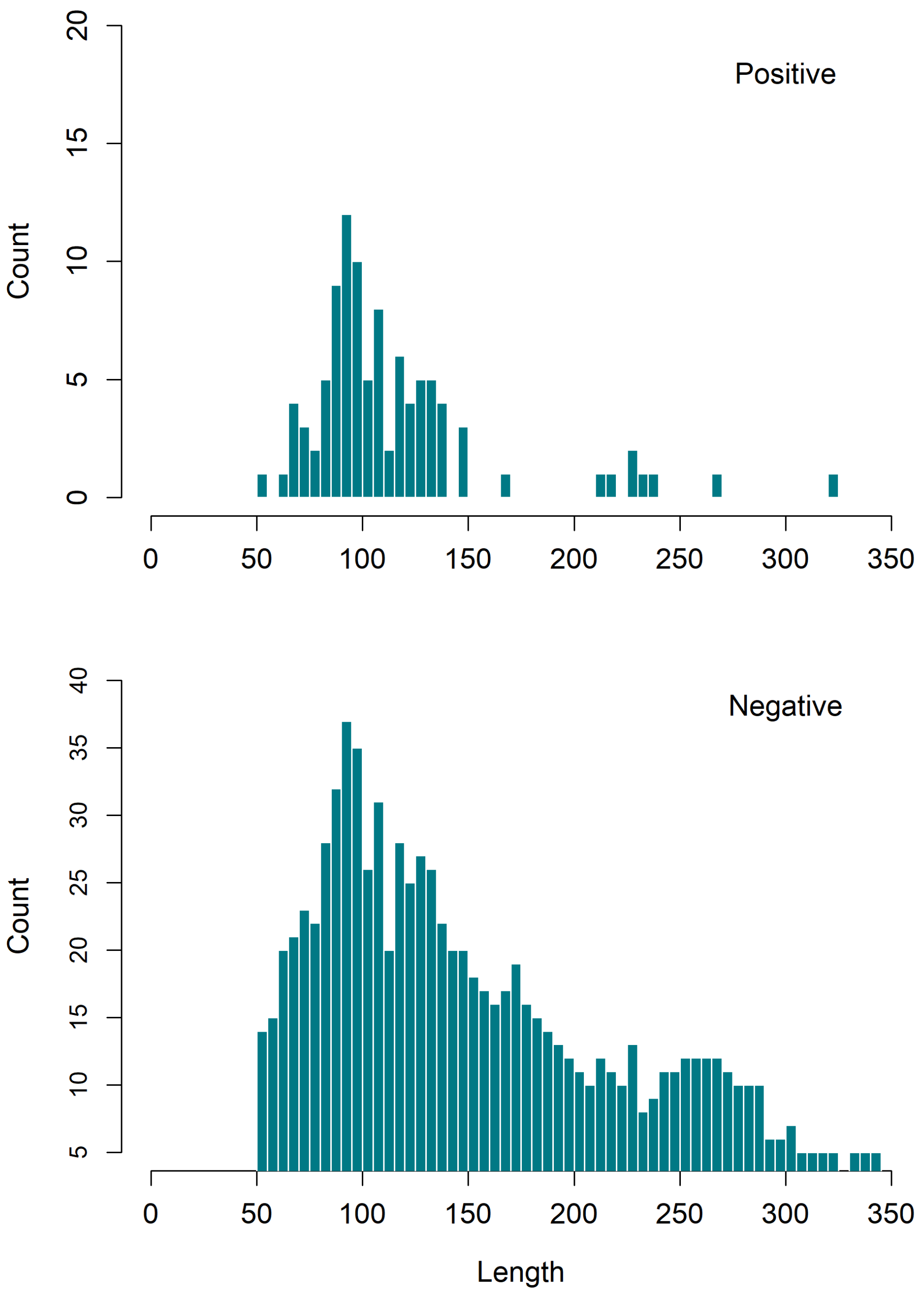

Fig. S5. Distributions of sequence lengths of the 98 anti-CRISPRs and 902 non-anti-CRISPRs from the training dataset. Non-anti-CRISPRs consist of 607 phage-derived proteins and 295 proteins derived from bacterial known and putative MGEs, provided that those bacterial genera were known to harbour anti-CRISPRs.

Table S2. Details of all 26 anti-CRISPRs used in the independent dataset.

ID	Name	Associated anti-CRISPR type	Bacterial origin	Similarity to training anti-CRISPRs	Reference
1	AcrID1	I-D	*Sulfolobus*	56.12%	(He, et al., 2018)
2	AcrIC1	I-C	*Moraxella*	<10%	(Marino, et al., 2018)
3	AcrIE5	I-E	*Pseudomonas*	<10%	(Marino, et al., 2018)
4	AcrIE6	I-E	*Pseudomonas*	<10%	(Marino, et al., 2018)
5	AcrIE7	I-E	*Pseudomonas*	<10%	(Marino, et al., 2018)
6	AcrIE4-IF7	I-E	*Pseudomonas*	<10%	(Marino, et al., 2018)
7	AcrIF11	I-F	*Pseudomonas*	<10%	(Marino, et al., 2018)
8	AcrIF12	I-F	*Pseudomonas*	<10%	(Marino, et al., 2018)
9	AcrIF13	I-F	*Moraxella*	<10%	(Marino, et al., 2018)
10	AcrIF14	I-F	*Moraxella*	<10%	(Marino, et al., 2018)
11	AcrIIA6	II-A	*Streptococcus*	<10%	(Hynes, et al., 2018)
12	AcrIIC4	II-C	*Haemophilus*	<10%	(Lee, et al., 2018)
13	AcrIIC5	II-C	*Simonsiella*	<10%	(Lee, et al., 2018)
14	AcrVA1	V-A	*Moraxella*	<10%	(Marino, et al., 2018)
15	AcrVA4	V-A	*Moraxella*	<10%	(Watters, et al., 2018)
16	AcrVA4_1	V-A	*Moraxella*	<10%	(Watters, et al., 2018)
17	AcrIIIB1	III-B	*Sulfolobus*	<10%	(Bhoobalan-Chitty, et al., 2019)
18	AcrIIA7	II-A	*Metagenomic libraries*	<10%	(Uribe, et al., 2019)
19	AcrIIA8	II-A	*Metagenomic libraries*	<10%	(Uribe, et al., 2019)
20	AcrIIA9	II-A	*Metagenomic libraries*	<10%	(Uribe, et al., 2019)
21	AcrIIA10	II-A	*Metagenomic libraries*	<10%	(Uribe, et al., 2019)
22	AcrIIA11	II-A	*Clostridium*	<10%	(Forsberg, et al., 2019)
23	AcrIIA12_1	II-A	*Listeria*	<10%	(Osuna, et al., 2019)

24	AcrIIA13	II-A	*Staphylococcus*	<10%	(Watters, et al., 2020)
25	AcrIIA12_2	II-A	*Staphylococcus*	21.38%	(Watters, et al., 2020)
26	AcrIIA12_3	II-A	*Staphylococcus*	<10%	(Watters, et al., 2020)

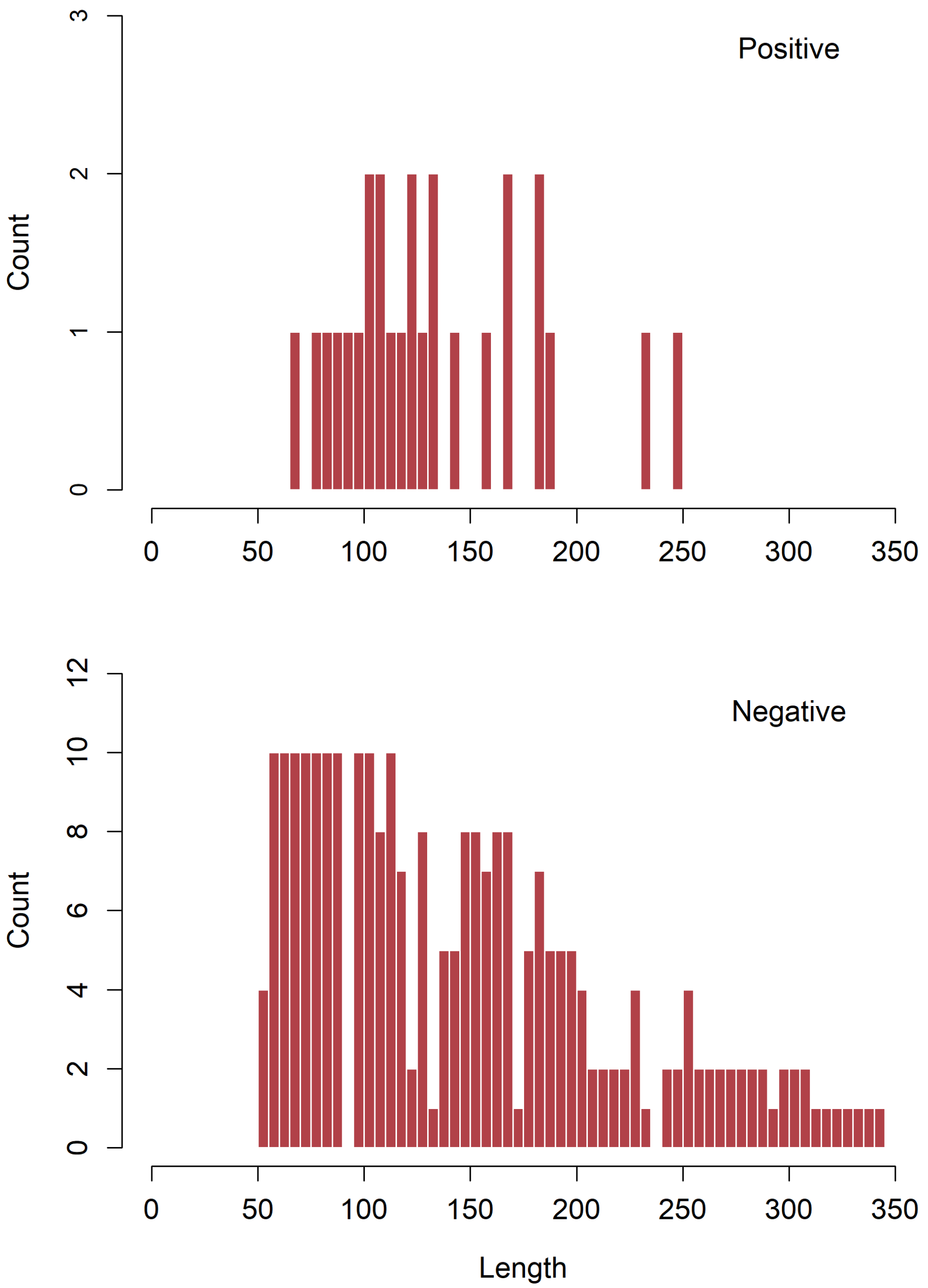

Fig. S6. Distributions of sequence lengths of 26 anti-CRISPRs and 260 non-anti-CRISPRs from the independent dataset. Non-anti-CRISPRs consist of 182 phage-derived proteins and 78 proteins derived from bacterial known and putative MGEs, provided that those bacterial genera were known to harbour anti-CRISPRs.

Table S3. Details of the five recently discovered anti-CRISPRs used in the case study.

ID	Name	Associated anti-CRISPR type	Bacterial origin	Reference
1	AcrIIA16*	II-A	*Listeria*	(Mahendra, et al., 2020)
2	AcrIIA17*	II-A	*Enterococcus*	(Mahendra, et al., 2020)
3	AcrIIA18	II-A	*Streptococcus*	(Mahendra, et al., 2020)
4	AcrIIA19	II-A	*Staphylococcus*	(Mahendra, et al., 2020)
5	AcrIII-1	III-A III-B	*Sulfolobus*	(Athukoralage, et al., 2020)

Note: *Not to be confused with the “AcrIIA16” and “AcrIIA17” that were predicted by AcRanker and later renamed to AcrIIA20 and AcrIIA21 respectively in their formally published paper.

Table S4. Prediction performance of single feature-based models, and the final ensemble model based on the 5-fold cross-validation test.

Encoding	SN	SP	ACC	F-value	MCC
AAC	0.753±0.027	0.765±0.053	0.758±0.031	0.753±0.029	0.518±0.065
DPC	0.801±0.036	0.742±0.036	0.771±0.026	0.774±0.026	0.546±0.054
PSSM-composition	0.852±0.018	0.845±0.031	0.850±0.024	0.847±0.022	0.697±0.046
DPC-PSSM	0.835±0.039	0.821±0.045	0.826±0.030	0.825±0.030	0.656±0.062
PSSM-AC	0.789±0.046	0.809±0.047	0.799±0.023	0.794±0.025	0.601±0.047
RPSSM	0.879±0.038	0.835±0.036	0.856±0.028	0.858±0.027	0.716±0.051
PaCRISPR	**0.909±0.013**	**0.856±0.028**	**0.882±0.017**	**0.883±0.016**	**0.765±0.031**

Note: Values appeared in the form of mean±standard deviation. The best performance value for each metric across different models is highlighted in bold. The final model of PaCRISPR integrates four PSSM-based models by averaging their prediction scores. This applies to all other results if not explicitly specified.

Table S5. Prediction performance of single feature-based models and the final ensemble model based on the independent test.

Encoding	SN	SP	ACC	F-value	MCC
AAC	0.731±0.000	0.777±0.102	0.754±0.051	0.750±0.041	0.512±0.110
DPC	0.692±0.000	0.688±0.272	0.690±0.136	0.702±0.091	0.389±0.284
PSSM-composition	0.769±0.000	**0.850±0.142**	0.810±0.071	0.805±0.058	0.628±0.150
DPC-PSSM	0.731±0.000	0.823±0.125	0.777±0.062	0.769±0.050	0.561±0.132
PSSM-AC	0.808±0.000	0.827±0.132	0.817±0.066	0.819±0.055	0.640±0.135
RPSSM	0.692±0.000	**0.850±0.152**	0.771±0.076	0.756±0.059	0.557±0.164
PaCRISPR	**0.846±0.000**	**0.850±0.139**	**0.848±0.069**	**0.851±0.058**	**0.702±0.138**

Note: Values appeared in the form of mean±standard deviation. The best performance value for each metric across different models is highlighted in bold.

Table S6. The detailed prediction results of 26 positive samples in the independent dataset, by the single feature-based models and the final ensemble model of PaCRISPR, compared with the BLAST-based baseline predictor and AcRanker. Samples that were predicted incorrectly (with the prediction scores less than 0.5 or with a symbol "-") are highlighted in grey. If the toolkits did not provide an exact or easily-discernable prediction score, then " √ " was used to denote that the anti-CRISPR was predicted to be an anti-CRISPR and "-" was used to denote that the anti-CRISPR was predicted to not be an anti-CRISPR.

ID	Name	Sequence-based feature		Evolutionary feature				PaCRISPR	BLAST	AcRanker
		AAC	DPC	PSSM-composition	DPC-PSSM	PSSM-AC	RPSSM			
1	AcrID1	0.946	0.973	0.959	0.966	0.965	0.947	0.959	√	√
2	AcrIC1	0.870	0.917	0.987	0.850	0.479	0.945	0.815	-	√
3	AcrIE5	0.791	0.738	0.703	0.523	0.582	0.768	0.644	-	√
4	AcrIE6	0.262	0.440	0.706	0.327	0.663	0.536	0.558	-	√
5	AcrIE7	0.485	0.534	0.650	0.605	0.727	0.627	0.652	-	-
6	AcrIE4-IF7	0.732	0.669	0.896	0.936	0.693	0.820	0.836	√	√
7	AcrIF11	0.504	0.615	0.327	0.782	0.680	0.688	0.620	-	√
8	AcrIF12	0.526	0.397	0.628	0.297	0.743	0.734	0.601	-	√
9	AcrIF13	0.618	0.641	0.952	0.777	0.521	0.927	0.794	-	-
10	AcrIF14	0.601	0.734	0.889	0.786	0.849	0.745	0.817	-	-
11	AcrIIA6	0.638	0.572	0.904	0.909	0.536	0.755	0.776	-	-
12	AcrIIC4	0.601	0.344	0.802	0.400	0.877	0.487	0.642	√	-
13	AcrIIC5	0.816	0.783	0.982	0.963	0.761	0.886	0.898	-	√
14	AcrVA1	0.743	0.551	0.852	0.537	0.476	0.489	0.588	-	√
15	AcrVA4	0.273	0.278	0.856	0.808	0.622	0.688	0.744	-	-
16	AcrVA4_1	0.607	0.785	0.856	0.913	0.667	0.714	0.787	-	√
17	AcrIIIB1	0.236	0.105	0.311	0.446	0.447	0.340	0.386	-	-
18	AcrIIA7	0.573	0.589	0.358	0.478	0.140	0.309	0.321	-	-
19	AcrIIA8	0.267	0.465	0.041	0.316	0.578	0.181	0.279	-	√
20	AcrIIA9	0.396	0.268	0.213	0.147	0.395	0.280	0.259	-	√
21	AcrIIA10	0.558	0.794	0.754	0.639	0.658	0.805	0.714	-	√
22	AcrIIA11	0.860	0.634	0.940	0.587	0.634	0.455	0.654	-	√

23	AcrIIA12_1	0.891	0.712	0.989	0.846	0.820	0.887	0.885	-	√
24	AcrIIA13	0.849	0.884	0.952	0.846	0.701	0.934	0.859	-	√
25	AcrIIA12_2	0.312	0.225	0.484	0.751	0.829	0.373	0.609	-	-
26	AcrIIA12_3	0.693	0.590	0.791	0.849	0.740	0.694	0.768	-	√

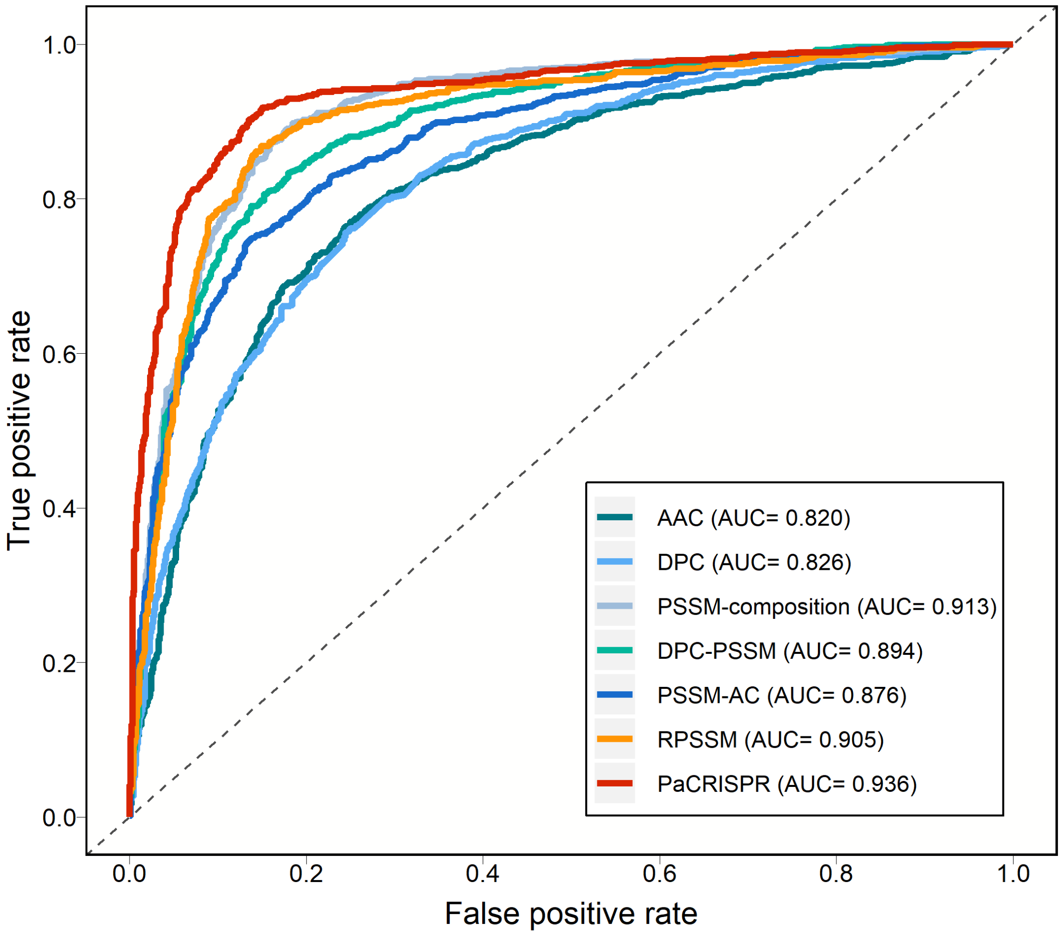

Fig. S7. ROC curves of single feature-based models, and the final ensemble model of PaCRISPR based on the 5-fold cross-validation test.

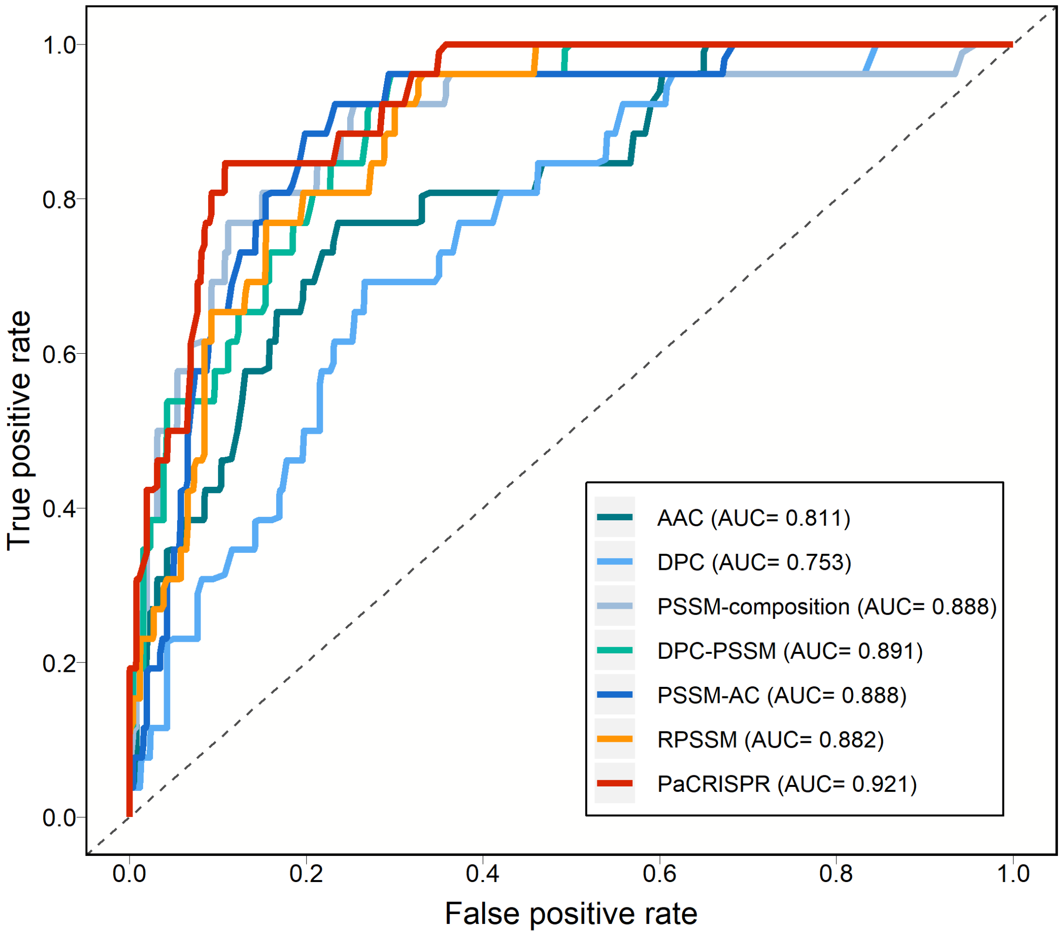

Fig. S8. ROC curves of single feature-based models, and the final ensemble model of PaCRISPR based on the independent test.

Table S7. Prediction performance comparison of PaCRISPR, AcRanker and the BLAST-based baseline predictor on the independent dataset.

Model	SN	SP	ACC	F-value	MCC
BLAST	0.115±0.000	**1.000±0.000**	0.558±0.000	0.207±0.000	0.247±0.000
AcRanker	0.654±0.000	0.769±0.140	0.712±0.070	0.697±0.051	0.432±0.149
PaCRISPR	**0.846±0.000**	0.850±0.139	**0.848±0.069**	**0.851±0.058**	**0.702±0.138**

Note: Values represent mean±standard deviation. The best performance value for each metric across these predictors is highlighted in bold.

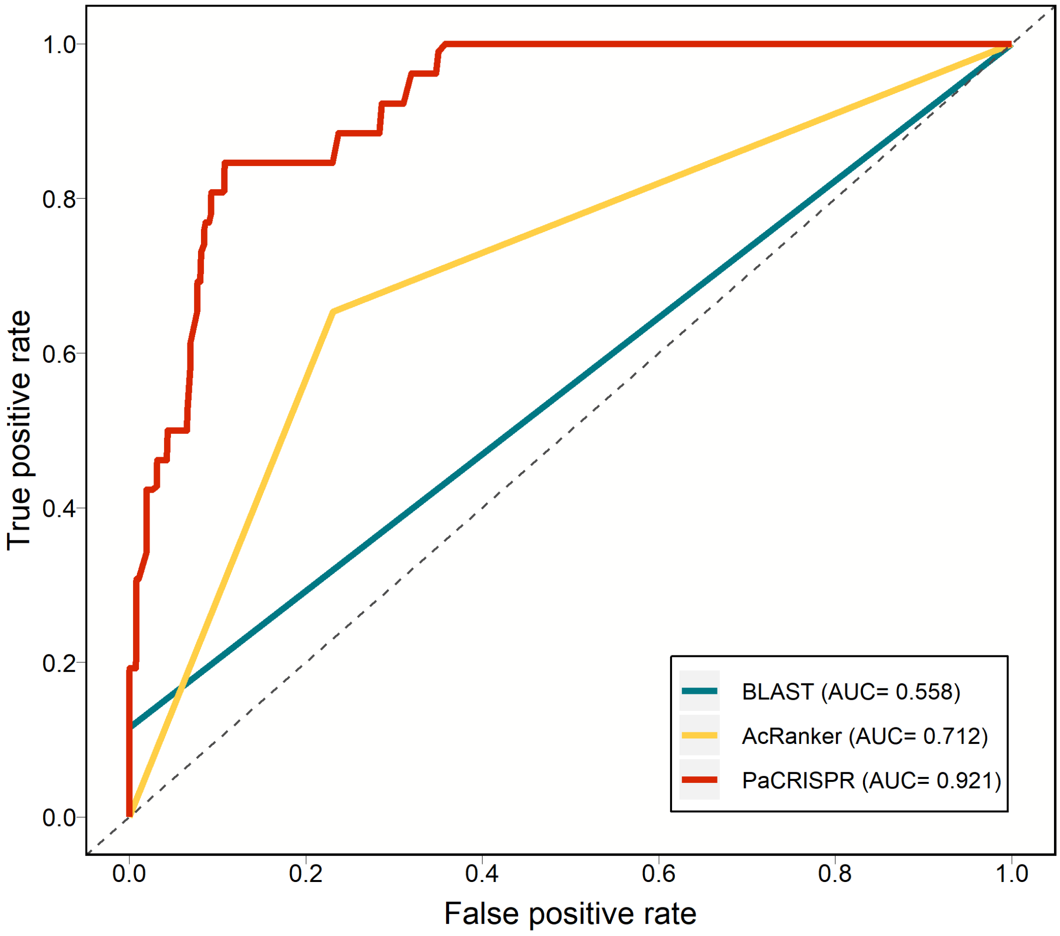

Fig. S9. ROC curves of PaCRISPR, AcRanker and the BLAST-based baseline predictor based on the independent dataset.

Table S8. Prediction comparison of PaCRISPR and AcRanker on two datasets of long non-anti-CRISPR proteins.

Non-anti-CRISPR proteins	Number of sequences	Minimal length	Maximal length	Number of correct predictions	
				AcRanker	PaCRISPR
Phage	266	351	3500	265	258
Bacterial MGE	597	350	989	595	596

Table S9. Comparison of ranked lists of a bacterial contig predicted by PaCRISPR and AcRanker. This contig (NCBI-RefSeq: NZ_ALTM01000002.1) contains a novel AcRanker-discovered anti-CRISPR (AcrIIA21), and is part of *Streptococcus agalactiae* strain GB00548. It was further expanded by another AcRanker-discovered anti-CRISPR (AcrIIA20) and all 31 anti-CRISPRs from the independent dataset and case study. The 33 known anti-CRISPRs, highlighted in green, act as markers to measure the performance of the predictors in practical use.

PaCRISPR			AcRanker		
Protein ID	Rank	Score	Protein ID	Rank	Score
AcrID1	1	0.959	AcrIIA20	1	-0.785
AcrIIC5	2	0.898	AcrIIA12_1	2	-2.146
AcrIIA12_1	3	0.885	WP_000078282.1_16	3	-2.360
AcrIIA13	4	0.859	AcrIC1	4	-2.709
AcrIE4-IF7	5	0.836	AcrIE5	5	-3.073
AcrIF14	6	0.817	AcrIIC5	6	-3.193
AcrIC1	7	0.815	AcrIIA13	7	-3.226
AcrIIA17	8	0.814	WP_000331953.1_26	8	-3.273
AcrIIA19	9	0.797	AcrIIA11	9	-3.382
AcrIF13	10	0.794	WP_000654333.1_10	10	-3.699
AcrVA4_1	11	0.787	AcrIIA9	11	-3.940
AcrIIA6	12	0.776	WP_000052405.1_11	12	-4.054
AcrIIA12_3	13	0.768	WP_000944235.1_50	13	-4.163
AcrVA4	14	0.744	WP_000259072.1_12	14	-4.211
AcrIIA10	15	0.714	AcrIIA12_3	15	-4.229
AcrIIA11	16	0.654	WP_000390802.1_9	16	-4.304
AcrIE7	17	0.652	WP_000259017.1_17	17	-4.336
AcrIE5	18	0.644	AcrVA4_1	18	-4.368
AcrIIC4	19	0.642	WP_000798242.1_76	19	-4.379
WP_000259072.1_12	20	0.639	AcrIE4-IF7	20	-4.395
WP_000204784.1_42	21	0.627	AcrID1	21	-4.433
AcrIF11	22	0.620	WP_000793595.1_33	22	-4.437
AcrIIA18	23	0.617	AcrIE6	23	-4.454
AcrIIA12_2	24	0.609	AcrIF12	24	-4.603
AcrIIA20	25	0.603	WP_000791272.1_69	25	-4.629
AcrIF12	26	0.601	AcrIIA17	26	-4.678
AcrVA1	27	0.588	WP_000384271.1_24 (AcrIIA21)	27	-4.776
WP_001220479.1_21	28	0.559	WP_001018249.1_44	28	-4.794
AcrIE6	29	0.558	AcrIII-1	29	-4.810
WP_000130119.1_19	30	0.553	AcrIIA8	30	-4.812
AcrIII-1	31	0.503	AcrVA1	31	-4.835
WP_001865978.1_59	32	0.442	WP_001865978.1_59	32	-4.913
WP_000390802.1_9	33	0.442	WP_001134681.1_13	33	-4.930
WP_000602921.1_8	34	0.431	WP_000568029.1_38	34	-4.934

WP_000654333.1_10	35	0.402	AcrIF11	35	-4.939
AcrIIA16	36	0.393	AcrIIA10	36	-4.939
AcrIIIB1	37	0.389	WP_000416607.1_64	37	-4.955
WP_000134666.1_25	38	0.386	WP_000660181.1_70	38	-5.005
WP_000331953.1_26	39	0.365	WP_000749955.1_15	39	-5.012
WP_000905674.1_74	40	0.357	WP_001097380.1_27	40	-5.012
WP_000591144.1_23	41	0.330	AcrIIC4	41	-5.015
WP_000749955.1_15	42	0.329	WP_001867157.1_34	42	-5.015
AcrIIA7	43	0.326	WP_100222817.1_5	43	-5.055
WP_001019849.1_90	44	0.321	AcrIF13	44	-5.087
WP_000043857.1_43	45	0.300	WP_000602921.1_8	45	-5.090
WP_000259017.1_17	46	0.297	AcrIF14	46	-5.095
WP_000656477.1_40	47	0.297	WP_001867089.1_81	47	-5.128
WP_000907191.1_88	48	0.295	AcrIIA19	48	-5.132
WP_000421240.1_22	49	0.294	WP_000656477.1_40	49	-5.175
AcrIIA8	50	0.281	AcrIIA16	50	-5.231
AcrIIA9	51	0.279	WP_000217076.1_94	51	-5.289
WP_000660181.1_70	52	0.259	WP_000134666.1_25	52	-5.300
WP_000578331.1_67	53	0.257	WP_000244259.1_82	53	-5.412
WP_000793595.1_33	54	0.253	WP_001220479.1_21	54	-5.422
WP_000591129.1_39	55	0.248	AcrIIA7	55	-5.426
WP_001867090.1_75	56	0.243	AcrIIA12_2	56	-5.436
WP_001281321.1_71	57	0.241	WP_001217846.1_95	57	-5.457
WP_000052405.1_11	58	0.238	WP_000591129.1_39	58	-5.465
ALTM01000002.1_prot_46	59	0.234	WP_000035940.1_30	59	-5.547
WP_000228729.1_87	60	0.231	WP_000384859.1_18	60	-5.549
WP_000047535.1_77	61	0.227	AcrIIA6	61	-5.566
WP_001867089.1_81	62	0.192	ALTM01000002.1_prot_46	62	-5.571
WP_001134681.1_13	63	0.190	AcrIE7	63	-5.581
WP_000384271.1_24 (AcrIIA21)	64	0.172	WP_000601792.1_68	64	-5.604
WP_001867157.1_34	65	0.164	WP_001269501.1_93	65	-5.621
WP_000203492.1_48	66	0.153	WP_000793380.1_83	66	-5.655
WP_000078282.1_16	67	0.153	WP_000716636.1_36	67	-5.669
WP_000384859.1_18	68	0.152	WP_000285373.1_86	68	-5.680
WP_000434617.1_53	69	0.149	WP_000601104.1_79	69	-5.685
WP_000798242.1_76	70	0.141	WP_000777424.1_91	70	-5.692
WP_000944235.1_50	71	0.135	WP_000905674.1_74	71	-5.698
WP_000471421.1_7	72	0.135	WP_000122836.1_20	72	-5.704
WP_100222817.1_5	73	0.131	WP_001867090.1_75	73	-5.707
WP_000601104.1_79	74	0.130	WP_000421240.1_22	74	-5.709
WP_000777424.1_91	75	0.128	WP_000471421.1_7	75	-5.719
WP_000601792.1_68	76	0.127	WP_000043857.1_43	76	-5.741
WP_001874060.1_80	77	0.122	WP_001024512.1_6	77	-5.755

WP_001018249.1_44	78	0.116		WP_000250808.1_85	78	-5.761
WP_000285373.1_86	79	0.111		WP_000529593.1_1	79	-5.788
WP_001008570.1_78	80	0.110		WP_017645945.1_31	80	-5.798
WP_000791272.1_69	81	0.110		WP_001019849.1_90	81	-5.811
WP_000683316.1_61	82	0.109		WP_001874060.1_80	82	-5.830
WP_000793380.1_83	83	0.106		WP_000282450.1_65	83	-5.836
WP_000914796.1_3	84	0.104		AcrIIA18	84	-5.854
WP_000539954.1_41	85	0.103		WP_011074703.1_14	85	-5.855
WP_000282450.1_65	86	0.100		WP_000578331.1_67	86	-5.876
WP_000416607.1_64	87	0.098		WP_000203492.1_48	87	-5.879
WP_000568029.1_38	88	0.098		WP_000204784.1_42	88	-5.881
WP_000677351.1_49	89	0.097		WP_000697630.1_62	89	-5.906
WP_011074703.1_14	90	0.093		WP_000677351.1_49	90	-5.907
WP_000008111.1_73	91	0.088		AcrIIIB1	91	-5.909
WP_000217076.1_94	92	0.084		WP_088203103.1_84	92	-5.920
WP_000244259.1_82	93	0.083		WP_001867096.1_72	93	-5.924
WP_000151014.1_52	94	0.082		AcrVA4	94	-5.931
WP_000160572.1_32	95	0.081		WP_000130119.1_19	95	-5.933
WP_001867096.1_72	96	0.080		WP_000022172.1_28	96	-5.942
WP_000035940.1_30	97	0.076		WP_000914796.1_3	97	-5.942
WP_000122836.1_20	98	0.074		WP_000923270.1_92	98	-5.943
WP_000421727.1_60	99	0.073		WP_001008570.1_78	99	-5.946
WP_017645945.1_31	100	0.072		WP_000151014.1_52	100	-5.947
WP_000186183.1_66	101	0.069		WP_000178019.1_37	101	-5.948
WP_000680645.1_57	102	0.068		WP_000683316.1_61	102	-5.951
WP_000923270.1_92	103	0.067		WP_000539954.1_41	103	-5.951
WP_000603397.1_56	104	0.066		WP_000421727.1_60	104	-5.954
WP_000697630.1_62	105	0.063		WP_000140979.1_55	105	-5.960
WP_001024512.1_6	106	0.063		WP_000247053.1_2	106	-5.960
WP_000171304.1_54	107	0.062		WP_000591144.1_23	107	-5.966
WP_000228178.1_51	108	0.062		WP_000160572.1_32	108	-5.980
WP_088203103.1_84	109	0.061		WP_000186183.1_66	109	-5.983
WP_000716636.1_36	110	0.060		WP_000434617.1_53	110	-5.983
WP_001217846.1_95	111	0.059		WP_000143135.1_35	111	-5.986
WP_001269501.1_93	112	0.058		WP_000472390.1_89	112	-5.989
WP_000247053.1_2	113	0.057		WP_000907191.1_88	113	-5.989
WP_079254677.1_4	114	0.056		WP_000228729.1_87	114	-5.989
WP_000143135.1_35	115	0.054		WP_000047535.1_77	115	-5.989
WP_001203827.1_47	116	0.052		WP_000008111.1_73	116	-5.989
WP_000140979.1_55	117	0.051		WP_001281321.1_71	117	-5.989
WP_000178019.1_37	118	0.050		WP_000930334.1_63	118	-5.989
WP_000250808.1_85	119	0.048		WP_000170504.1_58	119	-5.989
WP_000022172.1_28	120	0.047		WP_000680645.1_57	120	-5.989
WP_000529593.1_1	121	0.046		WP_000603397.1_56	121	-5.989

WP_000930334.1_63	122	0.044	WP_000171304.1_54	122	-5.989
WP_000170504.1_58	123	0.044	WP_000228178.1_51	123	-5.989
WP_000472390.1_89	124	0.041	WP_001203827.1_47	124	-5.989
WP_001097380.1_27	125	0.027	WP_079254677.1_4	125	-5.989

Table S10. Comparison of numbers of anti-CRISPRs identified by PaCRISPR and AcRanker in different ranking ranges.

Top rank	Number of anti-CRISPRs	
	AcRanker	PaCRISPR
Top 5	4	5
Top 10	7	10
Top 15	9	15
Top 20	11	19
Top 25	14	23
Top 30	18	26
Top 35	20	27
Top 40	21	29
Top 45	23	30
Top 50	26	31
Top 55	27	32
Top 60	28	32
Top 65	30	33
Top 70	30	33
Top 75	30	33
Top 80	30	33
Top 85	31	33
Top 90	31	33
Top 95	33	33
Top 100	33	33

Reference

Athukoralage, J.S., *et al.* An anti-CRISPR viral ring nuclease subverts type III CRISPR immunity. *Nature* 2020;577(7791):572-575.

Bhoobalan-Chitty, Y., *et al.* Inhibition of Type III CRISPR-Cas Immunity by an Archaeal Virus-Encoded Anti-CRISPR Protein. *Cell* 2019;179(2):448-458 e411.

Bondy-Denomy, J., *et al.* Bacteriophage genes that inactivate the CRISPR/Cas bacterial immune system. *Nature* 2013;493(7432):429-432.

Forsberg, K.J., *et al.* Functional metagenomics-guided discovery of potent Cas9 inhibitors in the human microbiome. *Elife* 2019;8.

He, F., *et al.* Anti-CRISPR proteins encoded by archaeal lytic viruses inhibit subtype I-D immunity. *Nat Microbiol* 2018;3(4):461-469.

Hynes, A.P., *et al.* Widespread anti-CRISPR proteins in virulent bacteriophages inhibit a range of Cas9 proteins. *Nat Commun* 2018;9(1):2919.

Hynes, A.P., *et al.* An anti-CRISPR from a virulent streptococcal phage inhibits Streptococcus pyogenes Cas9. *Nat Microbiol* 2017;2(10):1374-1380.

Lee, J., *et al.* Potent Cas9 inhibition in bacterial and human cells by new anti-CRISPR protein families. *bioRxiv* 2018:350504.

Mahendra, C., *et al.* Broad-spectrum anti-CRISPR proteins facilitate horizontal gene transfer. *Nat Microbiol* 2020;5(4):620-629.

Marino, N.D., *et al.* Discovery of widespread type I and type V CRISPR-Cas inhibitors. *Science* 2018;362(6411):240-242.

Osuna, B.A., *et al.* Listeria phages induce Cas9 degradation to protect lysogenic genomes. *bioRxiv* 2019:787200.

Pawluk, A., *et al.* Naturally Occurring Off-Switches for CRISPR-Cas9. *Cell* 2016;167(7):1829-1838 e1829.

Pawluk, A., *et al.* A new group of phage anti-CRISPR genes inhibits the type I-E CRISPR-Cas system of Pseudomonas aeruginosa. *mBio* 2014;5(2):e00896.

Pawluk, A., *et al.* Inactivation of CRISPR-Cas systems by anti-CRISPR proteins in diverse bacterial species. *Nat Microbiol* 2016;1(8):16085.

Rauch, B.J., *et al.* Inhibition of CRISPR-Cas9 with Bacteriophage Proteins. *Cell* 2017;168(1-2):150-158 e110.

Smargon, A.A., *et al.* Cas13b Is a Type VI-B CRISPR-Associated RNA-Guided RNase Differentially Regulated by Accessory Proteins Csx27 and Csx28. *Mol Cell* 2017;65(4):618-630 e617.

Uribe, R.V., *et al.* Discovery and Characterization of Cas9 Inhibitors Disseminated across Seven Bacterial Phyla. *Cell host & microbe* 2019;25(2):233-241 e235.

Watters, K.E., *et al.* Systematic discovery of natural CRISPR-Cas12a inhibitors. *Science* 2018;362(6411):236-239.

Watters, K.E., *et al.* Potent CRISPR-Cas9 inhibitors from Staphylococcus genomes. *Proceedings of the National Academy of Sciences of the United States of America* 2020;117(12):6531-6539.
